# Differences in circulating appetite-related hormone concentrations between younger and older adults: a systematic review and meta-analysis

**DOI:** 10.1007/s40520-019-01292-6

**Published:** 2019-08-20

**Authors:** Kelsie Olivia Johnson, Oliver Michael Shannon, Jamie Matu, Adrian Holliday, Theocharis Ispoglou, Kevin Deighton

**Affiliations:** 1 Institute for Sport, Physical Activity and Leisure, Leeds Beckett University, Leeds, LS6 3QS UK; 2 Human Nutrition Research Centre, Institute of Cellular Medicine, Newcastle University, Newcastle upon Tyne, NE2 4HH UK; 3 School of Clinical and Applied Science, Leeds Beckett University, Leeds, LS1 3HE UK; 4 Faculty of Medicine and Health, Leeds University, Leeds, LS2 9JT UK; 5 Institute for Sport, Physical Activity and Leisure, Leeds Beckett University, Fairfax Hall, Leeds, LS6 3QQ UK

**Keywords:** Appetite, Anorexia of ageing, Hunger, Energy intake

## Abstract

Ageing is associated with reduced appetite and energy intakes. However, the mechanisms underlying this phenomenon are not fully understood. This systematic review and meta-analysis quantified differences in circulating concentrations of appetite-related hormones between healthy older and younger adults. Six databases were searched through 12th June 2018 for studies that compared appetite-related hormone concentrations between older and younger adults. Data were pooled using random-effects meta-analysis and are presented as standardised mean difference (Hedges’ g) with 95% confidence intervals (95% CI). Thirty-five studies were included involving 710 older adults (mean ± SD; age: 73 ± 5 years) and 713 younger adults (age: 28 ± 7 years). Compared with younger adults, older adults exhibited higher fasted and postprandial concentrations of the anorectic hormones cholecystokinin (Fasted: SMD 0.41 (95% CI 0.24, 0.57); *p *< 0.001. Postprandial: SMD 0.41 (0.20, 0.62); *p *< 0.001), leptin [Fasted: SMD 1.23 (0.15, 2.30); *p *= 0.025. Postprandial: SMD 0.62 (0.23, 1.01); *p *= 0.002] and insulin [Fasted: SMD 0.24 (− 0.02, 0.50); *p *= 0.073. Postprandial: SMD 0.16 (0.01, 0.32); *p *= 0.043]. Higher postprandial concentrations of peptide-YY were also observed in older adults compared with younger adults [SMD 0.31 (− 0.03, 0.65); *p *= 0.075]. Compared with younger adults, older adults had lower energy intakes [SMD − 0.98 (− 1.74, − 0.22); *p *= 0.011], and lower hunger perceptions in the fasted [SMD − 1.00 (− 1.54, − 0.46); *p *< 0.001] and postprandial states [SMD − 0.31, (− 0.64, 0.02); *p *= 0.064]. Higher circulating concentrations of insulin, leptin, cholecystokinin and peptide-YY accord with reduced appetite and energy intakes in healthy older adults. Interventions to reduce circulating levels of these hormones may be beneficial for combatting the anorexia of ageing.

**Electronic supplementary material:**

The online version of this article (10.1007/s40520-019-01292-6) contains supplementary material, which is available to authorized users.

## Introduction

Currently, 962 million people worldwide are over the age of 60 years [1] and it is predicted that 22% of the global population will be 60 years and older by 2050 [2]. A high proportion of health care is spent on older adults and this is set to increase given the projected rise in this population. Therefore, improving health in older adults is a crucial public health priority.

The “anorexia of ageing” describes a reduction in appetite and energy intake in older adults. Current observations suggest that this phenomenon has a prevalence of 15–30% in independently living older adults, with higher incidence occurring in hospital and nursing home settings [3]. The anorexia of ageing represents an independent risk factor for frailty, incident disability, morbidity and mortality [3–5]. Consequently, developing a better understanding of the mechanisms underlying the anorexia of ageing is important in designing interventions to prevent further deterioration in quality of life.

Appetite is regulated physiologically by the neuroendocrine system [6], with multiple hormones implicated as mediators of hunger and satiety. Several hormones have been identified to exert appetite-suppressing effects, including peptide-YY (PYY), glucagon-like-peptide-1 (GLP-1), oxytomodulin, pancreatic polypeptide (PP), cholecystokinin (CCK), gastric inhibitory peptide (GIP), insulin and leptin. Alternatively, ghrelin represents the only peripheral peptide known to exert an orexigenic effect [6, 7]. Current research suggests that changes in appetite-related hormones may mediate the reductions in appetite and energy intake observed in older adults. However, conclusions are yet to be drawn due to inconsistent findings between studies, likely due to differences in methodologies and the hormones measured. For example, results from Giezenaar et al. [8] demonstrated higher plasma concentrations of CCK in older adults compared with younger adults; however, these concentrations did not differ between older and younger adults in the study of Winkels et al. [9].

A systematic evaluation of the literature is required to gain a clear understanding of which hormones exhibit altered concentrations between older and younger adults, as well as the direction and size of any effects. Thus, the purpose of this research was to provide a systematic review and meta-analysis of studies which compared circulating appetite-related hormone concentrations between metabolically healthy older and younger adults in the fasted and/or postprandial state, with no known co-morbidities or pre-existing medical conditions. Comparisons of appetite perceptions and energy intake between the groups were also included where available. Understanding these effects contributes to the identification of potential mechanistic targets for future interventions to reduce the anorexia of ageing.

## Methods

This systematic review and meta-analysis was performed in accordance with the preferred reporting items for systematic review and meta-analyses (PRISMA) guidelines [10] and was prospectively registered with the PROSPERO database (CRD42017083747).

### Literature search

PubMed and The Cochrane Library as well as MEDLINE, SPORTDiscus, PsycINFO and CINAHL, via EBSCOhost, were searched in December 2017, with an updated search on 12th June 2018. These databases were used to provide a thorough approach for searches to obtain the maximum number of relevant studies, including relevant data from studies in which exercise or psychological interventions were implemented. Keyword searches were based on the population (i.e., older adults) in combination with appetite-related hormones, and appetite perceptions and/or energy intake. The specific keywords and the full search strategy can be found in supplementary material 1. The search strategy provided a total of 328 combinations. Reference lists of eligible studies and review articles were also hand searched. If only the abstract or partial data were published, then the author was contacted for the full data set. No language or date of publication restrictions were applied during the searches.

### Inclusion criteria

For inclusion, studies were required to meet the following criteria; participants in the study were not noted to be smokers, pregnant, or have a history of diabetes, gastrointestinal, inflammatory, metabolic, cardiovascular, neurological or psychological disease(s). Studies were excluded if there was evidence to show the implementation of a diet to induce energy imbalance, the administration of a supplement or taking medication as this is shown to affect appetite. Studies which investigated participants under the age of 18 were excluded. Studies were included if they were published in peer-reviewed journals, or were available as published conference proceedings, theses or dissertations to minimise the effect of any potential publication bias.

All studies were required to contain at least one of the following measures of circulating appetite-related hormone concentrations: leptin, acylated ghrelin, total ghrelin, oxyntomodulin, PYY, GLP-1, PP, GIP, CCK and/or insulin, in distinct groups of older and younger adults within the same study. Any postprandial comparisons of appetite-related hormone concentrations where < 125 kJ was provided during feeding were excluded from the analysis. Studies which involved the infusion of a nutrient as the feeding intervention were included, however, studies involving the infusion of pharmacological products were excluded. In addition to these outcomes, appetite perceptions and/or energy intake data were extracted if provided in the included studies.

Two researchers (KJ and OS) independently assessed titles and abstracts of studies for inclusion and later compared notes to reach a mutual consensus. Researchers were not blinded to the articles. However, research has shown that blinding during study selection and data extraction had neither a clinically nor a statistically significant effect on the summary outcome [11]. Disagreements about the eligibility of any particular studies were resolved by a third reviewer (KD). Potential studies that could be included based on their title or abstract were retrieved in full text and reviewed against the inclusion/exclusion criteria independently by two researchers (KJ and OS) with a third researcher (KD) used to settle any disputes. In total, 35 studies met the inclusion criteria and were included in this meta-analysis (see flow chart in supplementary figure 1). For a variable to be included in the meta-analysis, a minimum of three studies measuring the respective variable were required to meet the inclusion criteria.

### Data abstraction

Data were extracted independently by two separate researchers (KJ and OS) into a standardised spreadsheet which included (1) characteristics of articles valid for review; (2) the critical appraisal tool to assess the quality of cross-sectional studies (AXIS) (3) outcome data suitable for successive analysis based on mean, SD and sample size. Researchers were not blinded to articles during this process, however, risk of bias can be effectively conducted under blinded conditions [12]. Additional data were collected for study design, participant characteristics, the duration of observations and number of blood samples taken, hormone analytical method, as well as the assessment method of energy intake.

Where values were only presented in figure form, authors were contacted to provide the full dataset. If the dataset could not be retrieved (*n *= 17) the figure was digitised using graph digitiser software (DigitizeIt, Germany) and the means and SD/SEM were measured manually at the pixel level to the scale provided on the figure. DigitizeIt has been shown to provide valid and reliable results which provide good agreement with raw data values [13]. If area under the curve values were reported rather than the mean values, the mean values were calculated by dividing the area under the curve scores by time duration.

### Study appraisal and quality assessment

The critical appraisal tool to assess the quality of cross-sectional studies (AXIS) [14] was independently assessed by two reviewers (KJ and OS). Each included study was systematically evaluated against a 17-item checklist to judge study quality and the possibility of bias. Reviewers answered “yes” or “no” to each question within the checklist. When insufficient detail was reported, the reviewers answered the question with “do not know”. Disagreements were initially resolved via discussion between two independent reviewers (KJ and OS), however, a third reviewer (KD) was consulted for any necessary dispute resolution. Due to the cross-sectional nature of study designs within this meta-analysis, questions 7, 13 and 14 were removed with regard to addressing non-responders. Studies were not excluded based on the results of the quality assessment, however, for transparency, the quality of included studies has been presented in supplementary table 26.

### Statistical analysis

Missing standard deviations were calculated from standard errors or confidence intervals (CI). Outcome measures were converted into the standardised mean difference (SMD) and expressed as Hedges’ *g* with 95% CI which were used as the summary statistic. Correction using Hedges’ *g* is believed to yield an unbiased estimate of effect size [15]. The SMD represents the size of the difference between groups relative to the variability observed in the groups. A random-effects meta-analysis was performed by KJ and KD using Comprehensive Meta-Analysis Software (version 3, Biostat, Englewood, NJ, USA). A random-effects model was employed for all analyses based on the assumption that heterogeneity would exist between included studies due to the variability in study designs [16].The inputted data included sample sizes and outcome measures with their respective standard deviations.

Interpretation of SMD values was as follows: < 0.20 as trivial, 0.20–0.39 as small, 0.40–0.80 as moderate and > 0.80 as large [17]. A positive ES value indicated that hormone concentrations were higher in older adults compared with younger adults. Heterogeneity between trials was assessed using the I-squared statistic, where 0–40% suggests heterogeneity might not be important, 30–60% may represent moderate heterogeneity, 50–90% may represent substantial heterogeneity and 75–100% represents substantial heterogeneity [18]. This measure of heterogeneity was complimented by also reporting the Tau-squared statistic and the Chi squared statistic. To examine whether any conclusions were dependent on a single study, sensitivity analyses were employed for each variable by repeating the analysis with each study omitted in turn.

Where data were available, subgroup meta-analysis was performed for feeding method (infusion vs. eating), hormone analytical method (RIA vs. ELISA) and energy intake assessment method (researcher measured vs. self-reported). Each subgroup was required to have a minimum of three studies.

### Exploration of small study effects

Small study effects were explored with funnel plots of SMD versus standard errors [19] and by quantifying Egger’s linear regression intercept. A large and statistically significant Egger’s statistic indicates the presence of small study effects.

## Results

### Overview

Supplementary figure 1 outlines the flowchart of study selection. In total, 35 studies met the inclusion criteria for the meta-analysis. All included studies had been published (or accepted for publication) in peer-reviewed scientific journals at the time of inclusion (see supplementary material 2 for article references). Within the 35 studies, there were 31 fasted and 25 postprandial comparisons that were included between older and younger adults. Comparisons were segregated into fasted (see supplementary table 1) and postprandial (see supplementary table 2) responses to differentiate between findings during these two distinct periods of appetite regulation. Fasted comparisons represent single time points obtained after an overnight fast, whereas postprandial comparisons represent means of multiple time points after feeding.

There were four studies that involved more than two age groups; consequently, the youngest and oldest age groups were extracted for these studies. Out of the 35 studies which assessed circulating appetite-related hormone concentrations, 11 compared energy intake between older and younger adults (supplementary table 3). One study obtained energy intake by the 24-hour recall method, six studies by ad libitum meals and four through food diaries lasting 3–5 days. All studies which reported postprandial comparisons provided standardised meals or infusions to participants with an energy content of 502–4064 kJ (mean: 2454 kJ) and had hormone observation periods of 0.5–24 h (mean: 4.41 h). All studies involving postprandial comparisons provided mixed macronutrient meals, with 19 studies providing it in solid form, 6 studies involving the infusion of a nutrient, 2 studies providing it in liquid form and 1 study comparing both liquid and solid form. Three studies manipulated the macronutrient composition of the mixed meals to identify if this dictated postprandial outcomes. If a study contained more than one postprandial comparison for each age group, then the results were combined with the study being the unit for analysis. The effects of macronutrient manipulation were conflicting between studies, therefore, it is difficult to draw conclusions on the effects of macronutrient distribution on differences in appetite-related hormone concentrations between older and younger adults.

Hunger was assessed alongside appetite-related hormone concentrations in 13 out of the 35 studies, with 7 studies evaluating both fasted and postprandial responses, 5 studies reporting only fasted responses and 1 study only presenting postprandial hunger perceptions between older and younger adults (supplementary material 4).

### Participant demographics

A total of 713 younger adults and 710 older adults were included in this meta-analysis. Gender was reported in 31 out of 35 studies (532 men and 573 women; 48.1% men). Mean age ranged from 19 to 50 (mean: 28) years for younger adults. For older adults, all studies ranged from 65 to 85 (mean: 73) years. Mean BMI was reported in 25 out of 35 studies and ranged from 21.2 to 28.1 (mean: 23.6) kg m^−2^ for younger adults and 20.5–26.9 (mean: 24.8) kg m^−2^ for older adults, therefore falling in the healthy weight category for both groups.

### Meta-analysis

Individual study statistics and results for each outcome variable are summarised in supplementary tables 5–22.

### Standardised mean difference for acylated ghrelin concentrations

Only one study which met the inclusion criteria included the measurement of circulating acylated ghrelin concentrations in the fasted state. Consequently, this could not be included in the meta-analysis.

Postprandial acylated ghrelin concentrations were lower in older adults than younger adults, with a small difference between groups (SMD: − 0.21, 95% CI: − 0.76 to 0.35; *n *= 5; *p *= 0.466; supplementary figure 2). The degree of heterogeneity may be moderate between studies (*I*^2^ = 52.6%; *Q* = 8.4, *τ*^2^ = 0.208, *d*_f_ = 4).

### Standardised mean difference for total ghrelin concentrations

There was little difference in fasted total ghrelin concentrations when comparing older adults with younger adults (SMD: 0.13, 95% CI: − 0.12 to 0.39; *n *= 6; *p *= 0.312; supplementary figure 3a). The degree of heterogeneity was found to be low between studies (*I*^2^ = 0.00%; *Q* = 5.0, *τ*^2^ < 0.0005, *d*_f_ = 5).

There was little difference in postprandial total ghrelin concentrations when comparing older adults with younger adults (SMD: 0.17, 95% CI: − 0.06 to 0.40; *n *= 8; *p *= 0.146; supplementary figure 3b). The degree of heterogeneity was found to be low between studies (*I*^*2*^ =0.0%, *Q* = 2.8, *τ*^2^ < 0.0005, *d*_f_ = 7).

### Standardised mean difference for CCK concentrations

**Fig. 1:**
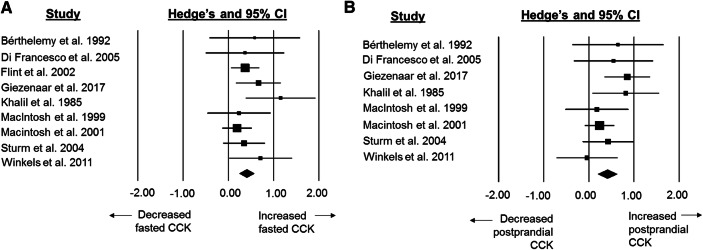
Forest plot of standardised mean differences (means ± 95% confidence intervals [CIs] for studies evaluating the differences in fasted CCK concentrations (**a**) and postprandial CCK concentrations (**b**) in older versus younger adults. The size of each square represents the relative weight of each comparison. The diamond represents the SMD (mean ± 95% CI) for the model

Fasted CCK concentrations were higher in older adults than younger adults with a moderate difference between groups (SMD: 0.41, 95% CI: 0.24 to 0.57; *n *= 9; *p *< 0.0005; Fig. 1a). The degree of heterogeneity was found to be low between studies (*I*^*2*^= 0.0%, *Q* = 7.6, *τ*^2^ < 0.0005, *d*_f_ = 8).

Postprandial CCK concentrations were higher in older adults than younger adults with a moderate difference between groups (SMD: 0.41, 95% CI: 0.20–0.62, *n *= 8; *p *< 0.0005; Fig. 1b). The degree of heterogeneity was found to be low between studies (*I*^2^= 7.2%, *Q* = 7.5, *τ*^2^ = 0.085, *d*_f_ = 7).

### Standardised mean difference and moderator variables for GLP-1 concentrations

There was little difference in fasted GLP-1 concentrations when comparing older adults with younger adults (SMD: − 0.02, 95% CI: − 0.44 to 0.40; *n *= 7; *p *= 0.942; supplementary figure 4a). The degree of heterogeneity may be substantial between studies (*I*^2^=75.8%; *Q* = 24.8, *τ*^2^ = 0.235 and *d*_f_ = 6).

There was little difference in postprandial GLP-1 concentrations when comparing older adults with younger adults (SMD: 0.11, 95% CI: − 0.19 to 0.41; *n *= 7; *p *= 0.474; supplementary figure 4b). The degree of heterogeneity was found to be moderate between studies (*I*^2^=48.7%, *Q* = 11.7, *τ*^2^ = 0.078, *d*_f_ = 6).

### Standardised mean difference for leptin concentrations

**Fig. 2:**
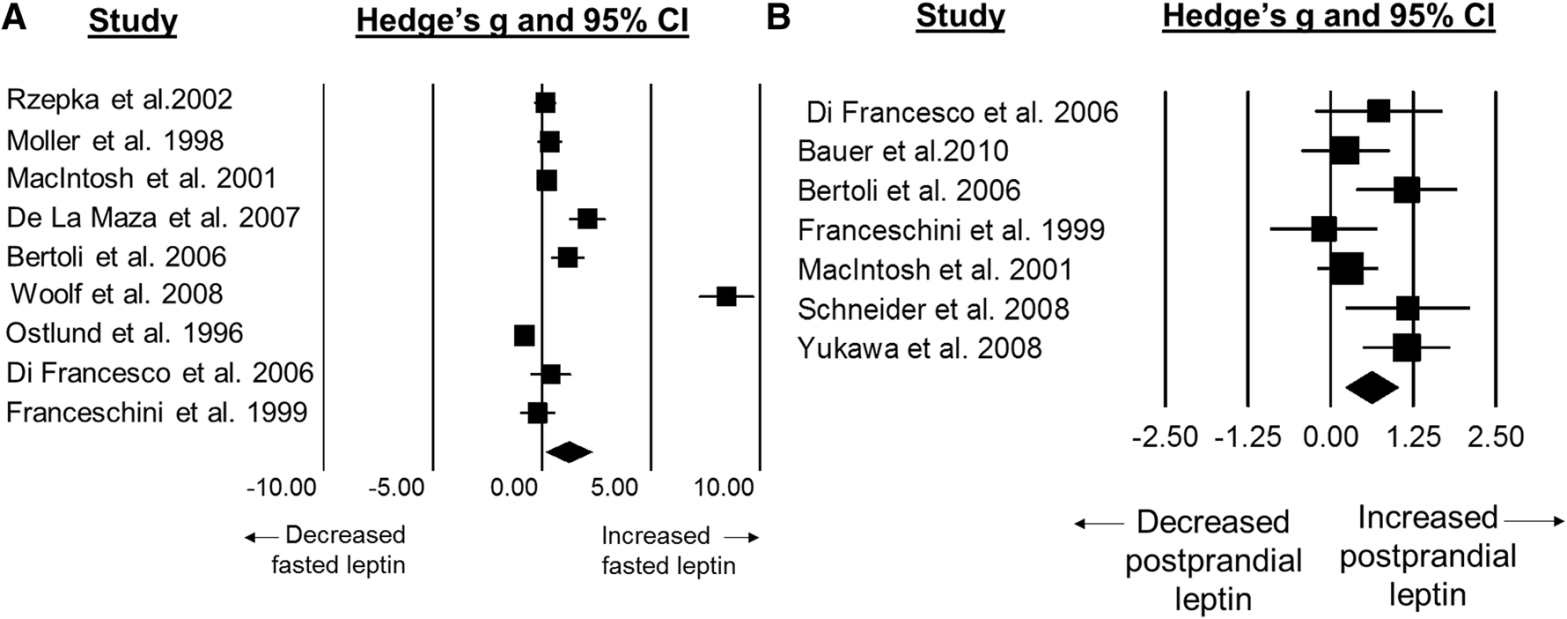
Forest plot of standardised mean differences (means ± 95% confidence intervals [CIs] for studies evaluating the differences in fasted leptin concentrations (**a**) and postprandial leptin concentrations (**b**) in older versus younger adults. The size of each square represents the relative weight of each comparison. The diamond represents the SMD (mean ± 95% CI) for the model

Fasted leptin concentrations were higher in older adults than younger adults with a large difference between groups (SMD: 1.23, 95% CI: 0.15 to 2.30; *n *= 9; *p *= 0.025; Fig. 2a). The degree of heterogeneity was found to be considerable between studies (*I*^2^=96.7%, *Q* = 238.9, *τ*^2^ = 2.545, *d*_f_ = 8).

Postprandial leptin concentrations were higher in older adults than younger adults with a moderate difference between groups (SMD: 0.62, 95% CI: 0.23 to 1.01; *n *= 7; *p *= 0.002; Fig. 2b). The degree of heterogeneity was found to be moderate between studies (*I*^2^=51.5%, *Q* = 12.4, *τ*^2^ = 0.138, *d*_f_ = 6).

### Standardised mean difference and moderator variables for insulin concentrations

**Fig. 3:**
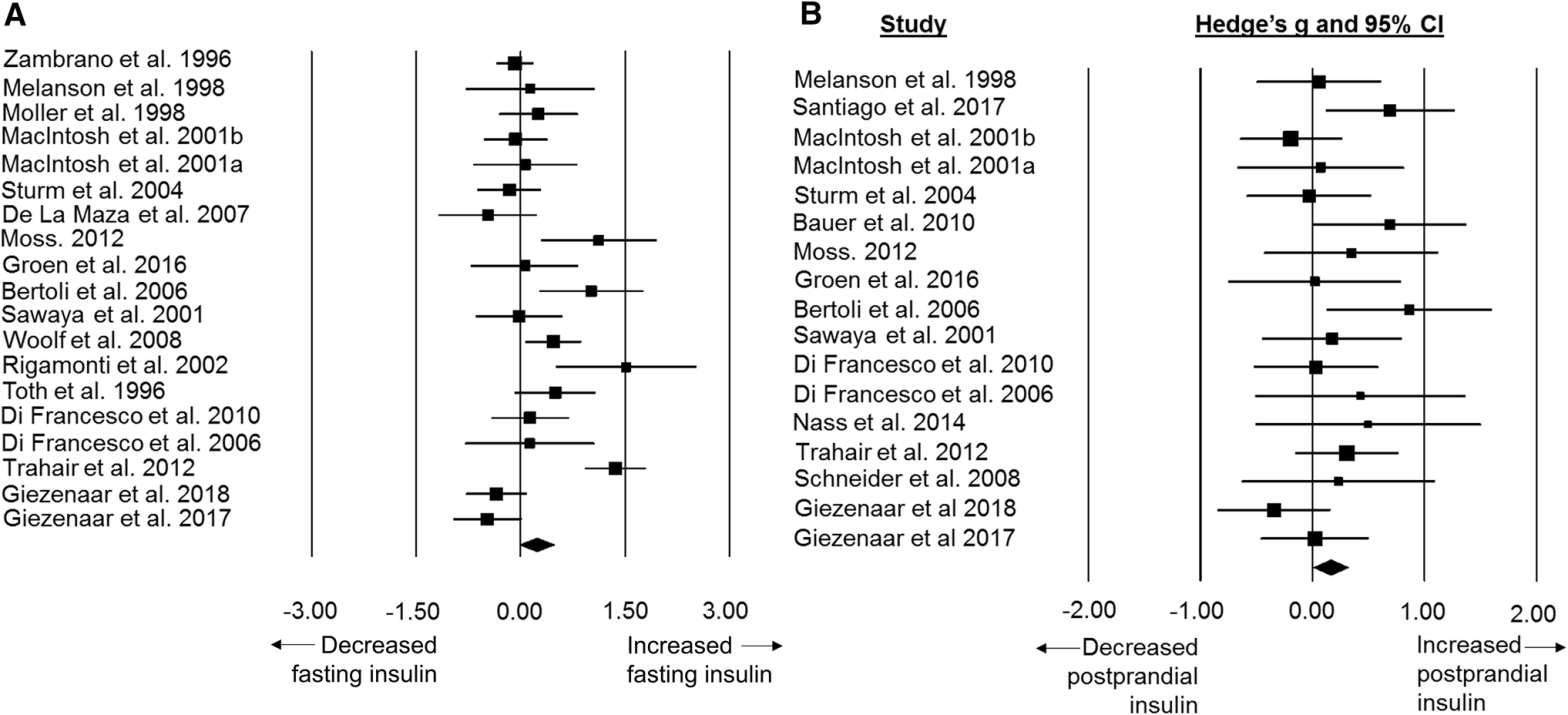
Forest plot of standardised mean differences (means ± 95% confidence intervals [CIs] for studies evaluating the differences in fasted insulin concentrations (**a**) and postprandial insulin concentrations (**b**) in older versus younger adults. The size of each square represents the relative weight of each comparison. The diamond represents the SMD (mean ± 95% CI) for the model

Fasted insulin concentrations were higher in older adults than younger adults with a small difference between groups (SMD: 0.24, 95% CI: − 0.02 to 0.50; *n *= 19; *p *= 0.073; Fig. 3a) The degree of heterogeneity may be substantial between studies (*I*^2^=74.3%, *Q* = 70.1, *τ*^2^ = 0.225, *d*_f_ = 18). Subgroup analysis revealed no significant effect of the hormone analytical method on outcomes (*p* = 0.99; Table 1).

**Table 1:** Summary of moderator variable analysis for energy intake, GLP-1 and insulin by subgroup

Moderator Variable	*p* value	Comparison
Hormone analytical method		
Fasted insulin	0.985	RIA (*n* = 10; SMD 0.26, 95% CI − 0.03 to 0.55) ELISA (*n* = 7; SMD 0.22, 95% CI − 0.35 to 0.80)
Postprandial insulin	0.438	RIA (*n* = 7; SMD 0.34, 95% CI 0.07 to 0.60) ELISA (*n* = 7; SMD 0.06, 95% CI − 0.19 to 0.31)
Type of feeding		
Postprandial insulin	0.427	Infusion (*n* = 3; SMD 0.01, 95% CI − 0.41 to 0.44) Feeding (*n* = 14; SMD 0.20, 95% CI 0.03 to 0.37)
Postprandial GLP-1	0.296	Feeding (*n* = 3; SMD 0.30; 95% CI − 0.12 to 0.73) Infusion (*n* = 4; SMD − 0.01, 95% CI − 0.41 to 0.39)
Energy intake assessment method		
Energy intake	0.015	Researcher measured (*n* = 7; SMD − 0.10, 95% CI − 0.88 to 0.68) Self-report (*n* = 4; SMD − 2.89, 95% CI − 4.99 to − 0.78)

There was a trivial but significant difference in postprandial insulin concentrations between younger and older adults (SMD: 0.16, 95% CI: 0.01 to 0.32, *n *= 17; *p *= 0.043; Fig. 3b).The degree of heterogeneity was found to be low between studies (*I*^2^= 10.5%, *Q* = 17.9, *τ*^2^ = 0.001, *d*_f_ = 17).Subgroup analysis revealed no significant effect of the hormone analytical method or feeding method on outcomes (both *p* > 0.42; Table 1).

### Standardised mean difference for PYY concentrations

**Fig. 4:**
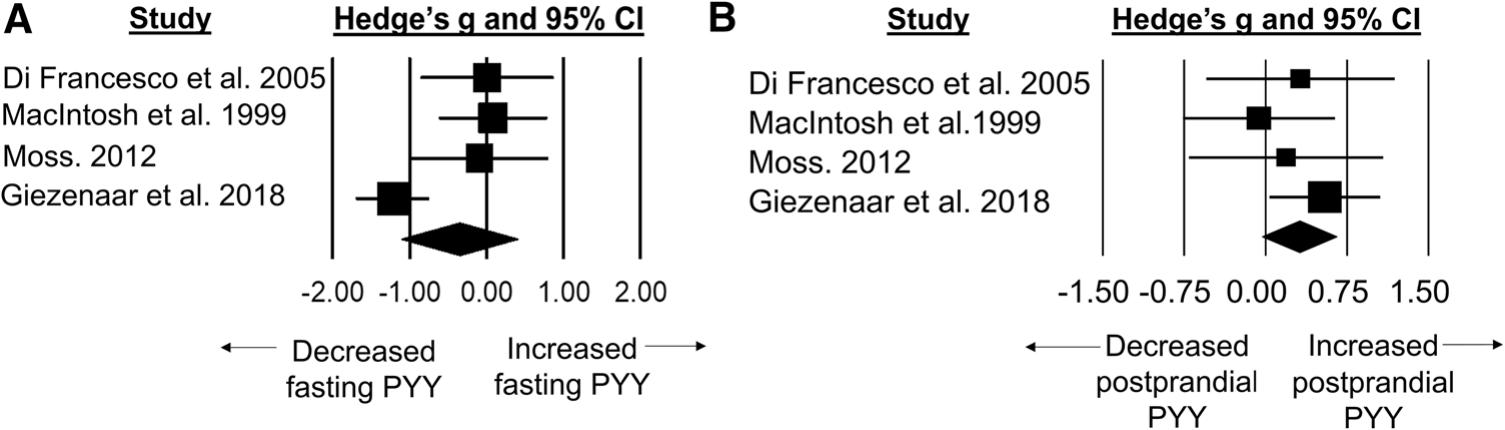
Forest plot of standardised mean differences (means ± 95% confidence intervals [CIs] for studies evaluating the differences in fasted PYY concentrations (**a**) and postprandial PYY concentrations (**b**) in older versus younger adults. The size of each square represents the relative weight of each comparison. The diamond represents the SMD (mean ± 95% CI) for the model

Fasted PYY concentrations were lower in older adults than younger adults with a small difference between groups (SMD: − 0.35, 95% CI: − 1.10 to 0.40, *n *= 4; *p *= 0.357; Fig. 4a). The degree of heterogeneity may be substantial between studies (*I*^2^=77.7%, *Q* = 13.4, *τ*^2^ = 0.445, *d*_f_ = 3).

Postprandial PYY concentrations were higher in older adults than younger adults with a small difference between groups (SMD: 0.31, 95% CI: − 0.03 to 0.65; *n *= 4; *p *= 0.075; Fig. 4b). The degree of heterogeneity was found to be low between studies (*I*^2^= 0.0%, *Q* = 2.0, *τ*^2^ < 0.0005, *d*_f_ = 3).

### Standardised mean difference and moderator variables for GIP concentrations

Fasted GIP concentrations were higher in older adults than younger adults with a small difference between groups (SMD: 0.16, 95% CI: − 0.23 to 0.54, *n *= 4; *p *= 0.334; supplementary figure 5a). The degree of heterogeneity was found to be substantial between studies (*I*^2^= 63.8%, *Q* = 8.3, *τ*^2^ = 0.102, *d*_f_ = 3).

Postprandial GIP concentrations were higher in older adults than younger adults with a small difference between groups (SMD: 0.25, 95% CI: − 0.19 to 0.70; *n *= 4; *p *= 0.263; supplementary figure 5b). The degree of heterogeneity was found to be substantial between studies (*I*^2^ = 68.3%, *Q* = 9.5, *τ*^2^ = 0.140, *d*_f_ = 3).

### Standardised mean difference and moderator variables for energy intake

**Fig. 5:**
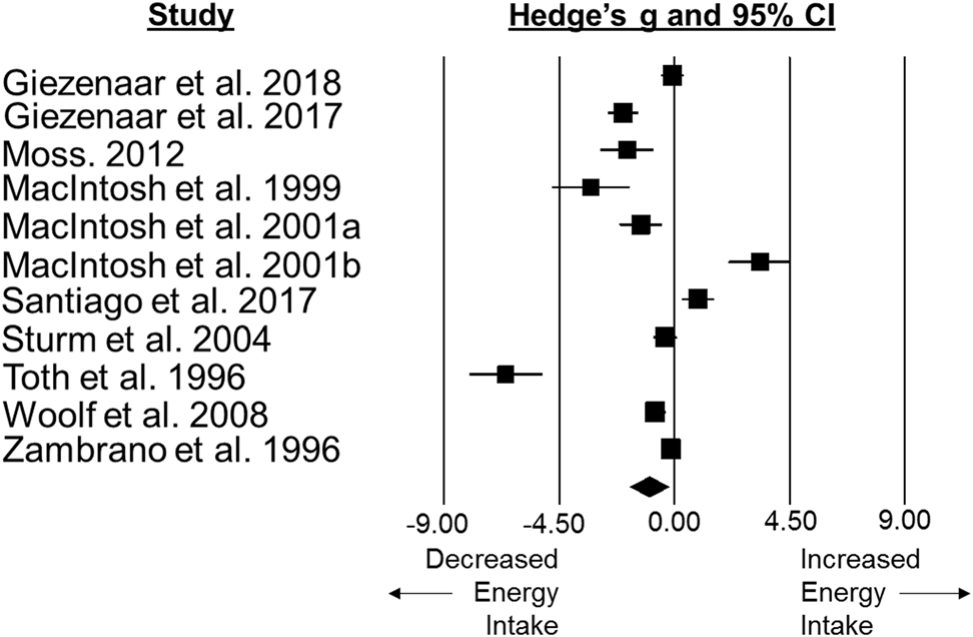
Forest plot of standardised mean differences (means ± 95% confidence intervals [CIs] for studies evaluating the differences in energy intake in older versus younger adults. The size of each square represents the relative weight of each comparison. The diamond represents the SMD (mean ± 95% CI) for the model

Energy intake was lower in older adults than younger adults with a large difference between groups (SMD: − 0.98, 95% CI: − 1.74 to − 0.22; *n *= 11; *p *= 0.011; Fig. 5). The degree of heterogeneity is substantial between studies (*I*^2^ = 94.6%; *Q* = 186.1, *τ*^2^ = 1.455, *d*_f_ = 10).). Subgroup analysis revealed a difference in energy intake between assessment types (*p *= 0.015), with older adults only showing a significantly lower energy intake compared with younger adults when assessed via self-report methods (Table 1).

### Standardised mean difference for hunger score

**Fig. 6:**
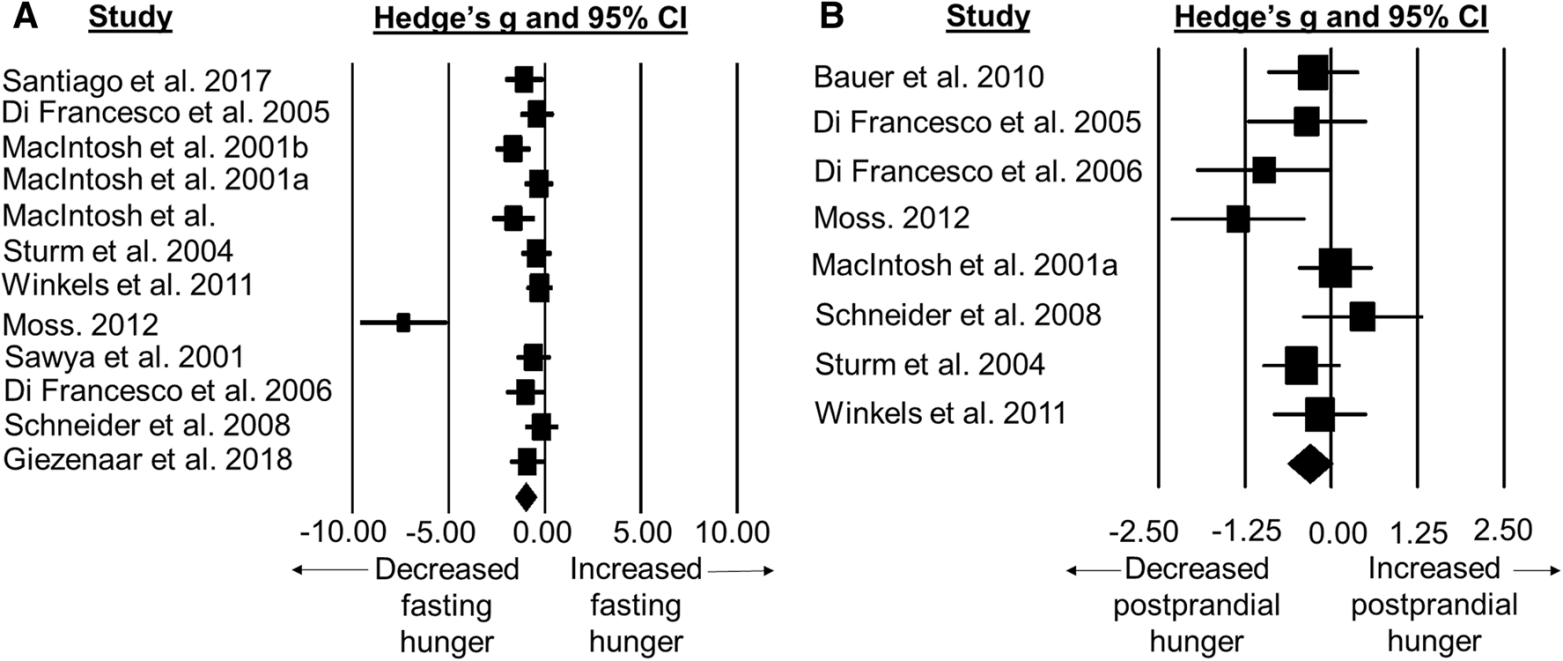
Forest plot of standardised mean differences (means ± 95% confidence intervals [CIs] for studies evaluating the differences in fasted hunger (**a**) and postprandial hunger (**b**) in older versus younger adults. The size of each square represents the relative weight of each comparison. The diamond represents the SMD (mean ± 95% CI) for the model

Fasted hunger scores were lower in older adults than younger adults with a moderate difference between groups (SMD: − 1.00, 95% CI: − 1.54 to − 0.46; *n *= 12; *p* = 0.0003; Fig. 6a). The degree of heterogeneity may be substantial between studies (*I*^2^= 76.1%; *Q* = 46.1, *τ*^2^ = 0.671, *d*_f_ = 11).

Postprandial hunger scores were lower in older adults than younger adults with a small difference between groups (SMD: − 0.31, 95% CI: − 0.64 to 0.02; *n *= 8; *p* = 0.064; Fig. 6b). The degree of heterogeneity may be moderate between studies (*I*^2^ = 37.7%; *Q* = 11.2, *τ*^2^ = 0.082, *d*_f_ = 7).

### Sensitivity analysis

Out of the 18 variables assessed in the meta-analysis, 11 variables revealed only minor changes when one study was omitted in turn from each analysis. The remaining seven variables revealed the removal of one or more comparison causing significant changes to the SMD (supplementary tables 23–25).

### Egger’s regression

5 out of the 18 variables assessed in the meta-analysis demonstrated some evidence of small study effects after inspection of the funnel plot and Egger’s regression intercept (see supplementary tables 23–25). The remainder of variables provided little evidence of small study effects.

### Quality assessment

The quality of studies appeared to vary. Most studies met more than 8 of the quality criteria items out of a possible 17 [mean (SD): 11 (2)]. All studies used the appropriate measurements to conduct assessment on appetite-related hormone concentrations. This included ELISA, RIA and standard chemiluminescent immunometric assays. The majority of studies included information regarding attaining ethical approval. However, the majority of studies failed to justify the sample size used or provide evidence for a power calculation. Some studies also lacked information on sources of recruitment and whether the sample represented the population investigated (Table 2). Reviewer evaluations matched on 88–100% of questions for each study. Since studies compared the difference between older and younger adults, the criteria regarding non-responders were not relevant (supplementary table 26).

**Table 2:** Criteria for quality assessment and number (%) of studies scoring points for each criterion

		Studies fulfilling the criteria, *n* (%)		
		Yes	No	Don’t know
Criteria				
1	Were the aims/objectives of the study clear?	21 (52.5)	19 (47.5)	0 (0.0)
Methods				
2	Was the study design appropriate for the stated aim(s)?	34 (85.0)	5 (12.5)	1 (2.5)
3	Was the sample size justified?	4 (10.0)	36 (90.0)	0 (0.0)
4	Was the target/reference population clearly defined? (is it clear who the research was about?)	26 (65.0)	12 (30.0)	2 (5.0)
5	Was the sample frame taken from an appropriate population base so that it closely represented the target/reference population under investigation?	12 (30.0)	3 (7.5)	25 (62.5)
6	Was the selection process likely to select subjects/participants that were representative of the target/reference population under investigation?	9 (22.5)	5 (5.0)	26 (65.0)
7	Were measures undertaken to address and categorise non-responders?	–	–	–
8	Were the risk factor and outcome variables measured appropriate to the aims of the study?	37 (92.5)	3 (7.5)	0 (0.0)
9	Were the risk factor and outcome variables measured correctly using instruments/measurements that had been trialled, piloted or published previously?	40 (100.0)	0 (0.0)	0 (0.0)
10	Is it clear what was used to determined statistical significance and/or precision estimates? (e.g., *p* values, confidence intervals)	27 (67.5)	13 (32.5)	0 (0.0)
11	Were the methods (including statistical methods) sufficiently described to enable them to be repeated?	20 (50.0)	20 (50.0)	0 (0.0)
Results				
12	Were the basic data adequately described?	23 (57.5)	17 (42.5)	0 (0.0)
13	Does the response rate raise concerns about non-response bias?	–	–	–
14	If appropriate, was information about non-responders described?	–	–	–
15	Were the results internally consistent?	32 (80.0)	7 (17.5)	1 (2.5)
16	Were the results presented for all the analyses described in the methods?	30 (75.0)	10 (25.0)	0 (0.0)
Discussion				
17	Were the authors’ discussions and conclusions justified by the results?	29 (72.5)	11 (27.5)	0 (0.0)
18	Were the limitations of the study discussed?	21 (52.5)	19 (47.5)	0 (0.0)
Other				
19	Were there any funding sources or conflicts of interest that may affect the authors’ interpretation of the results?	27 (67.5)	3 (7.5)	10 (25.0)
20	Was ethical approval or consent of participants attained?	38 (95.0)	0 (0.0)	2 (5.0)

## Discussion

Understanding differences in appetite-related hormone concentrations between older and younger adults provides mechanistic insight into the effect of ageing on the appetite-regulatory system. This systematic review and meta-analysis revealed higher fasted and postprandial circulating concentrations of CCK, insulin and leptin in older adults. We also observed higher postprandial concentrations of PYY in older versus younger adults. These findings were concomitant with reduced hunger and energy intake in older adults, which is in accordance with the anorectic effects of these hormones [6]. Alternatively, there were no clear differences between older and younger adults for circulating concentrations of GLP-1, GIP, total ghrelin and acylated ghrelin. The results from the present study suggest even without the presence of known metabolic diseases or co-morbidities, older adults experience alterations in appetite regulation.

The increase in CCK observed in the present review supports previous speculation that CCK signalling is increased during ageing. Indeed, the anorectic effects from elevated concentrations of CCK in the fasted and postprandial state may even be enhanced in older adults due to increased sensitivity to the actions of this hormone [20, 21] via CCK1 receptors on the nucleus of the solitary tract and vagal signalling [22]. In the postprandial state, CCK is released from the intestine in response to nutrients in the intestinal lumen and appears to be, at least partly, responsible for meal termination [23]. In addition to increased fasted satiating signals to the vagal nerve, increased circulating CCK also delays the rate of gastric emptying which is commonly seen in older adults. It has been suggested that delays in gastric emptying in older adults could reflect increased small intestinal feedback as a result of increased sensitivity and/or exposure of small intestine receptors to CCK [24]. Elevated postprandial concentrations and signalling of CCK may play a further role in enhanced satiation in combination with reductions in the gastric emptying rates of older adults.

The most robust differences between older and younger adults in the current review were observed for circulating concentrations of leptin, which were higher in older adults in both the fasted and postprandial state, with a large and moderate effect size, respectively. Leptin is an anorectic hormone secreted by adipose tissue [25] and therefore circulates in proportion to body fat stores as a tonic regulator of energy balance [26]. Consequently, the higher levels of leptin in older adults may be due to greater adiposity. However, adiposity is unlikely to be the sole mechanism for these elevations in leptin, as Zamboni et al. [27] demonstrated that after the adjustment for body mass, fasted levels of plasma leptin increased with age in a population of older women aged 67–78 years. It remains unclear whether older adults experience leptin resistance in association with increased circulating concentrations, as experienced by obese populations [28]. Consequently, understanding leptin sensitivity in older adults is important to appreciate whether elevated concentrations of this hormone exert an anorectic effect and whether leptin represents a therapeutic target for improving appetite outcomes in this population.

In accordance with the higher levels of leptin in older adults, fasted and postprandial insulin concentrations were also elevated. Circulating levels of insulin are determined primarily by peripheral insulin sensitivity [29] which alongside leptin correlate with adiposity [30] and deliver information on peripheral energy stores to the central nervous system. Although the primary role of insulin is related to metabolic regulation, it has also been shown to exert anorectic effects by acting on the arcuate nucleus of the hypothalamus to signal satiety and reduce food intake [31]. The observed increase in insulin may therefore contribute towards the attenuation of hunger in older adults observed in this review. However, as with leptin, insulin resistance of the hypothalamus may occur in older adults [32], thereby hindering the anorectic effects of this peptide. The relative importance of ageing and altered body composition as mediators of the increased insulin concentrations is unclear. The mean BMI value for younger (23.6 kg m^−2^) and older adults (24.8 kg m^−2^) was similar, suggesting that differences observed in insulin concentration are not a result of differences in weight status (as a proxy of body composition). In addition, despite differences in fasted insulin concentrations between older and younger adults, mean fasted plasma glucose concentrations were similar at 5.3 mmol L^−1^ and 5.0 mmol L^−1^, respectively, and suggested a healthy glucose tolerance in both groups. Additionally, mean HOMA-IR values for participants in the included studies were 1.67 for older adults and 1.57 for younger adults. Regardless, these findings highlight the need for interventions to reduce circulating concentrations of insulin from both an appetite and metabolic health perspective.

The PYY findings from this meta-analysis demonstrated a strong trend towards statistical significance for higher postprandial concentrations in older versus younger adults. This effect was observed despite the inclusion of only four studies and the subsequent likelihood of the analysis being underpowered. Interestingly, out of the four included studies in the present review, Moss [33] and Di Francesco et al. [34] both showed significantly higher circulating concentrations of PYY in older adults following the ingestion of a test meal. However, following intraduodenal infusion of lipid and glucose [35] and whey protein [36], circulating postprandial PYY concentrations were comparable between younger and older adults. Consequently, the effects of age on PYY concentrations appear to be most prominent when nutrients are naturally ingested, thereby including mastication and swallowing, rather than directly reaching the distal gastrointestinal tract [37]. It is possible that the observed results in the present review support previous speculations that bypassing of the stomach during intraduodenal infusion may attenuate PYY release, thereby diminishing any potential appetite-regulatory effects [7]. Critically, the ingestion of food as standardised test meals reflects typical human eating behaviours which suggests that increased PYY concentrations are likely to be experienced by older adults in response to habitual feeding. Unlike leptin and insulin, obesity does not seem to cause peripheral resistance to PYY [38–40], suggesting that the observed increases in older adults are likely to exert anorectic effects. The increased circulating PYY concentrations may further promote satiety by also contributing to reductions in gastric emptying rates in older adults [24].

Surprisingly, acylated ghrelin, total ghrelin, GLP-1 and GIP concentrations were not significantly different between older and younger adults in the present review. Alternatively, the differences in leptin, CCK, PYY and insulin appear to align with the reduction in hunger and energy intake in older adults observed in this meta-analysis. Consequently, it seems most beneficial for future interventions to target CCK, leptin, PYY and insulin when investigating methods to augment energy intake to reduce the anorexia of ageing from an endocrine perspective.

As a part of the present review, we extracted and analysed data for hunger perceptions and energy intake when measured alongside appetite-related hormones in the included studies. These findings mirror those of the recent meta-analysis by Giezenaar et al. [41] by demonstrating that energy intake was significantly lower in older adults than younger adults. However, in the present review, energy intake appeared to be moderated by the method of dietary assessment. Specifically, the results revealed that a difference in energy intake was only observed when self-report methods were used and not through researcher-assessed methods. It is apparent within the research literature that there is generalised underreporting when using self-report methods to assess energy intake. Therefore, our findings should be considered with caution as it is clear that limitations are present in self-report methods [42]. Nonetheless, the results indicate large decrease in fasted hunger in older adults compared with younger adults which is a known predictor for energy intake [43]. Postprandial hunger perceptions also tended to be lower in older adults than younger adults but with only a small effect size. In combination with differences in appetite-related hormone concentrations, these effects may also be mediated by diminished homeostatic regulation of physiological functions in older adults. For example, older adults have been shown to experience delayed gastric emptying rate, altered pyloric motility and increased antral area [34, 35, 44, 45].

In the current review, the hormones investigated which aligned with observed reductions in hunger and energy intake in older adults were CCK, leptin, insulin and PYY. It must be stressed that hormones involved in appetite regulation are only one aspect of a complex system that regulates feeding behaviour and that there may be other factors interacting which could also contribute to the changes in hunger and energy intake observed. Nevertheless, interventions to reduce circulating concentrations of these anorectic hormones may be useful to oppose the anorexia of ageing.

Despite the important findings in the current meta-analysis, some notable limitations must be acknowledged. First the hormones PP, oxyntomodulin and fasted acylated ghrelin are known to be mediators of appetite regulation. However, these hormones could not be included in the analysis due to only one study meeting the criteria for fasted acylated ghrelin and the absence of any studies measuring oxyntomodulin or PP concentrations. The present study explored differences in appetite regulation in healthy older adults, and it is therefore appreciated that the findings cannot be generalised to older adults who possess underlying co-morbidities. It must also be noted that the presence of co-morbidities and taking medication amongst participants was reported in only 22 studies, respectively. Consequently, the authors cannot be certain that all participants were free of underlying co-morbidities and were not taking medication. Additionally, despite our extensive search retrieving 3103 records, we cannot guarantee that our search was completely exhaustive of the relevant literature. However, having hand searched the reference lists of all included studies and review articles, we are confident that we have included all available relevant studies.

## Conclusions

This meta-analysis reveals that circulating concentrations of insulin, leptin, CCK and PYY are increased in older adults. Given the anorectic effect of these appetite-related hormones, such changes in older adults provides an underlying mechanism which may contribute towards the anorexia of ageing. Interventions to reduce circulating levels of these hormones may be beneficial for increasing appetite in older adults to attenuate, delay, or prevent the anorexia of ageing.

## Electronic supplementary material

Below is the link to the electronic supplementary material.

Supplementary material 1 (DOCX 509 kb)

Supplementary material 2 (DOCX 35 kb)

Supplementary material 3 (DOCX 20 kb)

Supplementary material 4 (DOCX 20 kb)

Supplementary material 5 (DOCX 57 kb)

Supplementary material 6 (DOCX 39 kb)

Supplementary material 7 (DOCX 33 kb)

Supplementary material 8 (DOCX 199 kb)
