# Supplementary material for: Differences in circulating appetite-related hormone concentrations between younger and older adults: a systematic review and meta-analysis
Source: Aging Clin Exp Res. 2019 Aug 20;32(7):1233–44. doi: 10.1007/s40520-019-01292-6 (PMC7316693; doi:10.1007/s40520-019-01292-6)
Supplement: Supplementary file 1 — Supplementary material 1 (DOCX 509 kb) [file 40520_2019_1292_MOESM1_ESM.docx]

**Article Title:** Differences in circulating appetite-related hormone concentrations between older and younger adults: a systematic review and meta-analysis

**Journal:** Aging Clinical and Experimental Research

**Author Names:** Kelsie Olivia Johnson, Oliver Michael Shannon, Jamie Matu, Adrian Holliday, Theocharis Ispoglou, Kevin Deighton

**Corresponding Author:** Dr Kevin Deighton, Institute for Sport, Physical Activity and Leisure, Leeds Beckett University, Leeds, LS6 3QS, United Kingdom (email: K.Deighton@leedsbeckett.ac.uk)


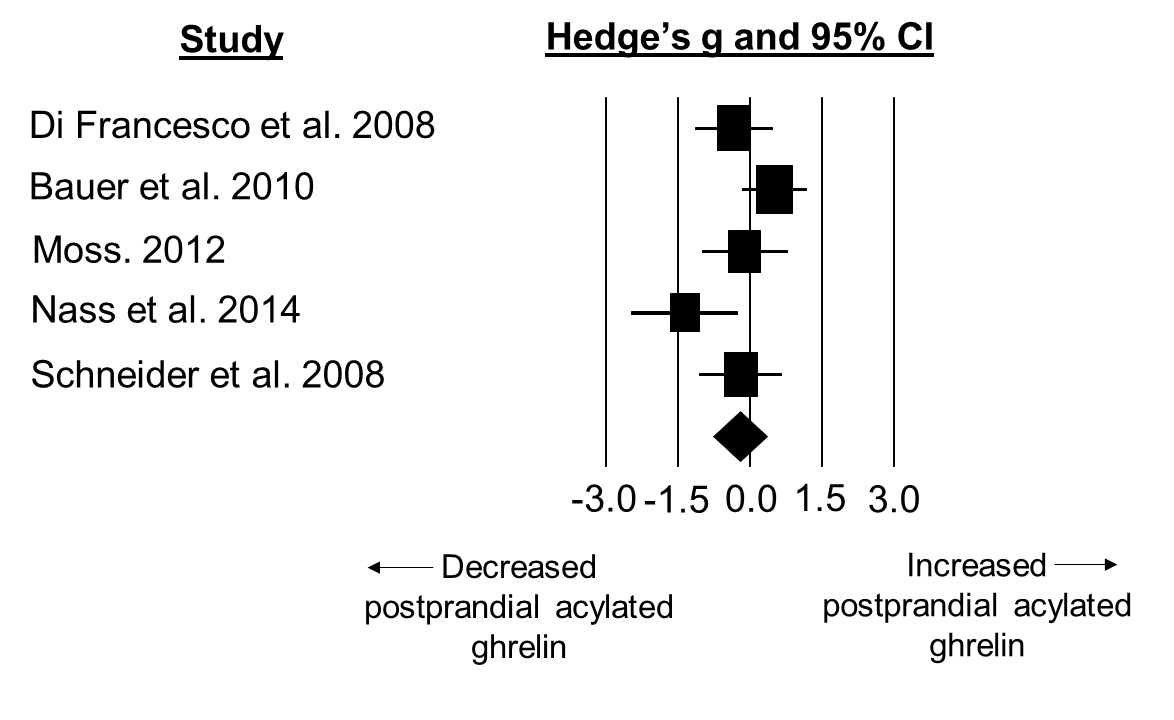


**Supplementary Figure 2.** Forest plot of standardised mean differences (means ± 95% confidence intervals [CIs]) for studies evaluating postprandial acylated ghrelin concentrations in older versus younger adults. The size of each square represents the relative weight of each comparison. The diamond represents the standardised mean difference (mean ± 95% CI) for the model.


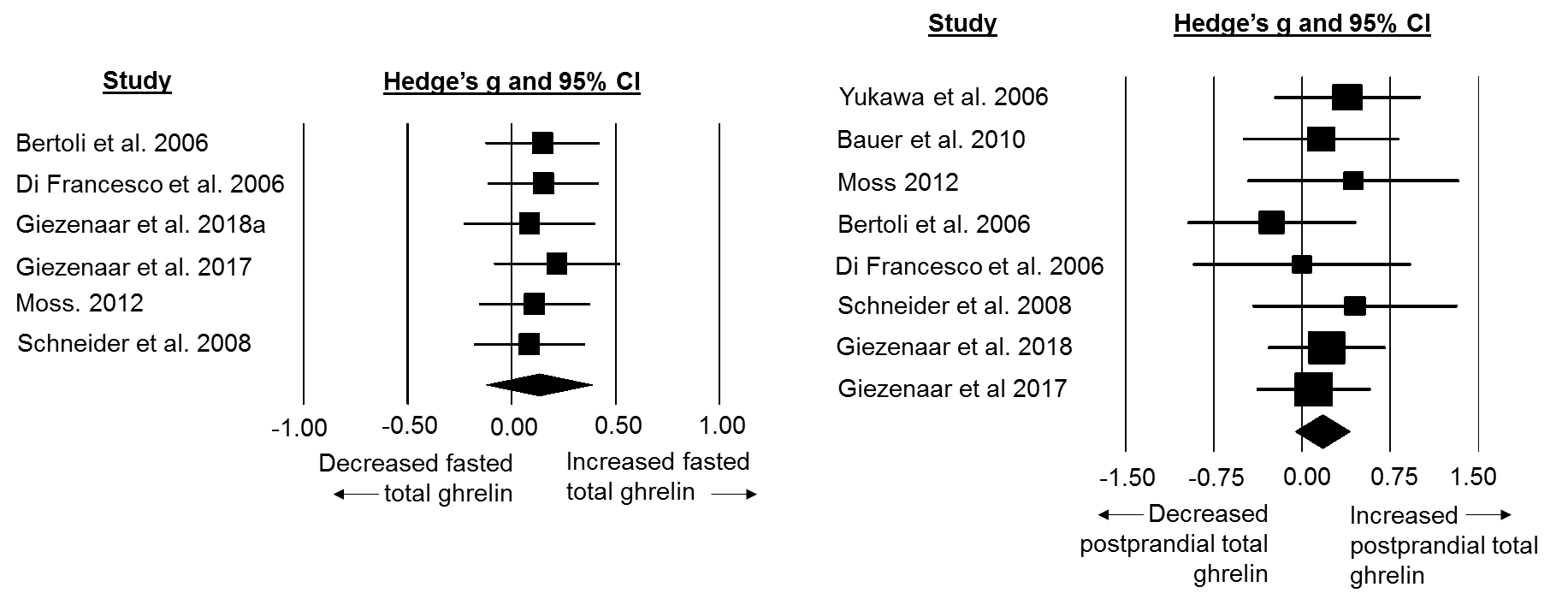


**Supplementary Figure 3**. Forest plot of standardised mean differences (means ± 95% confidence intervals [CIs]) for studies evaluating fasted total ghrelin concentrations (A) and postprandial total ghrelin concentrations (B) in older versus younger adults. The size of each square represents the relative weight of each comparison. The diamond represents the standardised mean difference (mean ± 95% CI) for the model.


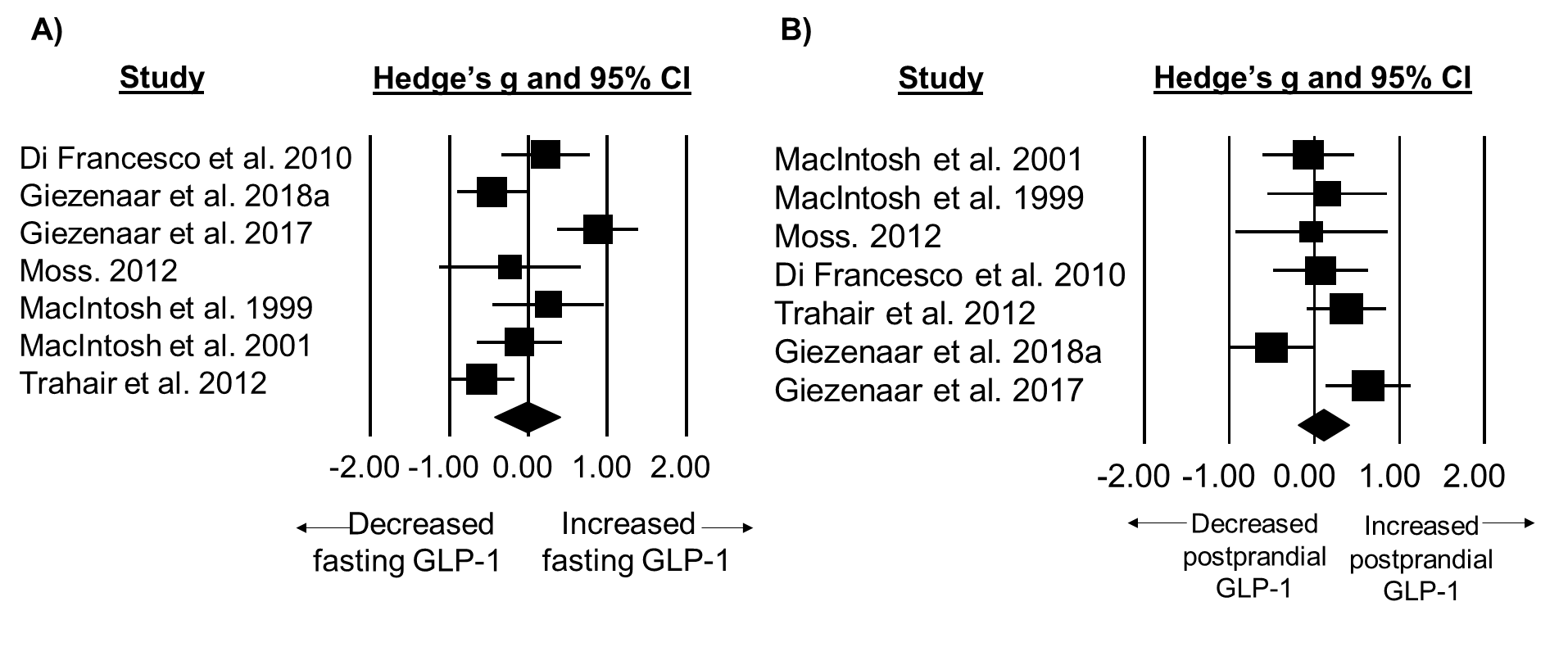


**Supplementary Figure 4.** Forest plot of standardised mean differences (means ± 95% confidence intervals [CIs]) for studies evaluating (A) fasted GLP-1 concentrations and (B) postprandial GLP-1 concentrations in older versus younger adults. The size of each square represents the relative weight of each comparison. The diamond represents the standardised mean difference (mean ± 95% CI) for the model.


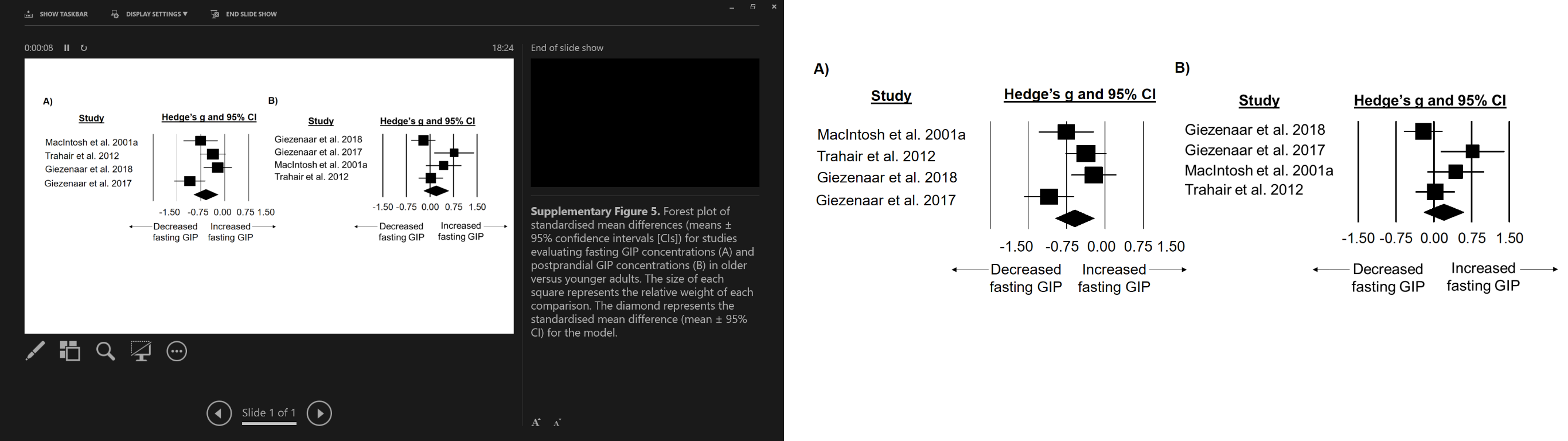


**Supplementary Figure 5.** Forest plot of standardised mean differences (means ± 95% confidence intervals [CIs]) for studies evaluating fasting GIP concentrations (A) and postprandial GIP concentrations (B) in older versus younger adults. The size of each square represents the relative weight of each comparison. The diamond represents the standardised mean difference (mean ± 95% CI) for the model.
