# Supplementary material for: Differences in circulating appetite-related hormone concentrations between younger and older adults: a systematic review and meta-analysis
Source: Aging Clin Exp Res. 2019 Aug 20;32(7):1233–44. doi: 10.1007/s40520-019-01292-6 (PMC7316693; doi:10.1007/s40520-019-01292-6)
Supplement: Supplementary file 2 — Supplementary material 2 (DOCX 35 kb) [file 40520_2019_1292_MOESM2_ESM.docx]

**Article Title:** Differences in circulating appetite-related hormone concentrations between older and younger adults: a systematic review and meta-analysis

**Journal:** Aging Clinical and Experimental Research

**Author Names:** Kelsie Olivia Johnson, Oliver Michael Shannon, Jamie Matu, Adrian Holliday, Theocharis Ispoglou, Kevin Deighton

**Corresponding Author:** Dr Kevin Deighton, Institute for Sport, Physical Activity and Leisure, Leeds Beckett University, Leeds, LS6 3QS, United Kingdom (email: K.Deighton@leedsbeckett.ac.uk)

**Supplementary Figure 1.** Flowchart of study selection. CCK: cholecystokinin; PP: pancreatic polypeptide; GLP-1: glucagon-like-peptide 1; GIP: gastric inhibitory peptide; PYY: peptide YY

Record identified through database screening

(n=3103)

Records Identified through other sources

(n=14)

Records Removed as duplicates

(n= 1569)

Records Screened

(n= 1548)

Full-text articles assessed for eligibility

(n= 64)

Studies included in quantitative synthesis (meta analysis)

(n=35)

19: Evaluating Fasting Insulin

19: Evaluating fasting leptin

19: Evaluating fasting CCK

7: Evaluating fasting GLP-1

6: Evaluating fasting total ghrelin

4: Evaluating fasting GIP

4: Evaluating fasting PYY

12: Evaluating fasting hunger

17: Evaluating postprandial insulin

7: Evaluating postprandial leptin

8: Evaluating postprandial CCK

7: Evaluating postprandial GLP-1

8: Evaluating postprandial total ghrelin

4: Evaluating postprandial GIP

4: Evaluating postprandial PYY

8: Evaluating postprandial hunger

11: Evaluating energy intake

Full-text articles excluded, with reasons why

(n=20)

2: No young vs old comparison

5: History of Disease

5: Smokers

2: Dieting

1: Altitude

2: Pharmalogical drug which influence appetite

3: Does not measure appetite-related hormones

5: Older group were not >65

Records excluded

(n= 1474)

84: Animal study

742: No Young vs Old Comparison

Cross-sectional

336: History of Disease

1: Participants aged <18

176: Does not measure appetite related hormone

2: Smokers

1: Dieters

132: Review

Certain data unobtainable from included studies

(n=2)

1: Evaluating Insulin

1: Evaluating CCK

Records excluded as unable to obtain data from author

(n=4)

1: Evaluating CCK

2: Evaluating Insulin

1: Evaluating PP
