# Supplementary material for: Differences in circulating appetite-related hormone concentrations between younger and older adults: a systematic review and meta-analysis
Source: Aging Clin Exp Res. 2019 Aug 20;32(7):1233–44. doi: 10.1007/s40520-019-01292-6 (PMC7316693; doi:10.1007/s40520-019-01292-6)
Supplement: Supplementary file 3 — Supplementary material 3 (DOCX 20 kb) [file 40520_2019_1292_MOESM3_ESM.docx]

**Article Title:** Differences in circulating appetite-related hormone concentrations between older and younger adults: a systematic review and meta-analysis

**Journal:** Aging Clinical and Experimental Research

**Author Names:** Kelsie Olivia Johnson, Oliver Michael Shannon, Jamie Matu, Adrian Holliday, Theocharis Ispoglou, Kevin Deighton

**Corresponding Author:** Dr Kevin Deighton, Institute for Sport, Physical Activity and Leisure, Leeds Beckett University, Leeds, LS6 3QS, United Kingdom (email: K.Deighton@leedsbeckett.ac.uk)

**Supplementary Material 1**

Search Strategy

The following terms were searched for in titles/abstracts of each database, with MeSH terms utilised where appropriate.

1. older people
2. older persons
3. older person
4. older individuals
5. older individual
6. older adults
7. older adult
8. aged
9. aged [MeSH Terms]
10. elderly
11. aging [MeSH Terms]
12. aging
13. ageing
14. appetite hormones
15. appetite hormone
16. appetite-related hormones
17. appetite-related hormone
18. appetite-regulating hormones
19. appetite-regulating hormone
20. gastrointestinal hormones
21. gastrointestinal hormone
22. GI hormones
23. GI hormone
24. Cholecystokinin
25. Cholecystokinin [MeSH Terms]
26. CCK
27. Peptide YY
28. PYY
29. Glucagon-like-peptide-1
30. Glucagon like peptide 1
31. Glucagon-like peptide 1 [MeSH Terms]
32. Glucagon-like peptide 1
33. Glucagon-like peptide 1
34. Glucagon-like-peptide 1
35. Glp 1
36. Glp-1
37. Leptin
38. Leptin [MeSH Terms]
39. Ghrelin
40. Ghrelin [MeSH Terms]
41. Pancreatic polypeptide
42. Gastric inhibitory peptide
43. Gastric-inhibitory-peptide
44. GIP
45. Oxyntomodulin
46. Oxyntomodulin [MeSH Terms]
47. OXM
48. Insulin
49. Insulin [MeSH Terms]
50. appetite
51. appetite [MeSH Terms]
52. hunger
53. hunger [MeSH Terms]
54. fullness
55. satiation
56. satiation [MeSH Terms]
57. satiety
58. desire to eat
59. prospective food consumption
60. energy intake
61. food intake
62. dietary intake
63. animals
64. humans

| **1** | **2** | **3** | **4** |
| --- | --- | --- | --- |
| **Study Population** | **Appetite Related Hormones** | **Subjective Appetite Sensations** | **Ad-Lib intake** |
| older people  older persons  older person  older individuals  older individual  older adults  older adult  aged  aged [MeSH Terms]  elderly  aging [MeSH Terms]  aging  ageing | appetite hormones  appetite hormone  appetite-related hormones  appetite-related hormone  appetite-regulating hormones  appetite-regulating hormone  gastrointestinal hormones  gastrointestinal hormone  GI hormones  GI hormone  Cholecystokinin  Cholecystokinin [MeSH Terms]  CCK  Peptide YY  PYY  Glucagon-like-peptide-1  Glucagon like peptide 1  Glucagon-like peptide 1 [MeSH Terms]  Glucagon-like peptide 1  Glucagon-like peptide 1  Glucagon-like-peptide 1  Glp 1  Glp-1  Leptin  Leptin [MeSH Terms]  Ghrelin  Ghrelin [MeSH Terms]  Pancreatic polypeptide  Gastric inhibitory peptide  Gastric-inhibitory-peptide  GIP  Oxyntomodulin  Oxyntomodulin [MeSH Terms]  OXM  Insulin  Insulin [MeSH Terms] | appetite  appetite [MeSH Terms]  hunger  hunger [MeSH Terms]  fullness  satiation  satiation [MeSH Terms]  satiety  desire to eat  prospective food consumption | energy intake  food intake  dietary intake |

1. older people or older persons or older person or older individuals or older individual or older adults or older adult or aged[ MeSH Terms] or elderly or aging[MeSH Terms] or aging or ageing

AND

1. appetite hormones or appetite hormone or appetite-related hormones or appetite-related hormone or appetite-regulating hormones or appetite-regulating hormone or gastrointestinal hormones or gastrointestinal hormone or GI hormones or GI hormone or Cholecystokinin or Cholecystokinin [MeSH Terms] or CCK or Peptide YY or PYY or Glucagon-like-peptide-1 or Glucagon like peptide 1 or Glucagon-like peptide 1 [MeSH Terms] or Glucagon-like peptide 1 or Glucagon-like peptide 1 or Glucagon-like-peptide 1 or Glp 1 or Glp-1 or Leptin or Leptin [MeSH Terms] or Ghrelin or Ghrelin [MeSH Terms] or Pancreatic polypeptide or Gastric inhibitory peptide or Gastric-inhibitory-peptide or GIP or Oxyntomodulin or Oxyntomodulin [MeSH Terms] or OXM or Insulin or Insulin [MeSH Terms]

AND

1. appetite or appetite [MeSH Terms] or hunger or hunger [MeSH Terms] or fullness or satiation or satiation [MeSH Terms] or satiety or desire to eat or prospective food consumption

OR

1. energy intake or food intake or dietary intake

NOT

1. animals [MeSH Terms] NOT humans [MeSH Terms]
