# Supplementary material for: Differences in circulating appetite-related hormone concentrations between younger and older adults: a systematic review and meta-analysis
Source: Aging Clin Exp Res. 2019 Aug 20;32(7):1233–44. doi: 10.1007/s40520-019-01292-6 (PMC7316693; doi:10.1007/s40520-019-01292-6)
Supplement: Supplementary file 4 — Supplementary material 4 (DOCX 20 kb) [file 40520_2019_1292_MOESM4_ESM.docx]

**Article Title:** Differences in circulating appetite-related hormone concentrations between healthy older and younger adults: a systematic review and meta-analysis

**Journal:** Aging Clinical and Experimental Research

**Author Names:** Kelsie Olivia Johnson, Oliver Michael Shannon, Jamie Matu, Adrian Holliday, Theocharis Ispoglou, Kevin Deighton

**Corresponding Author:** Dr Kevin Deighton, Institute for Sport, Physical Activity & Leisure, Leeds Beckett University, Leeds, LS6 3QS, United Kingdom (email: K.Deighton@leedsbeckett.ac.uk)

**Supplementary Material 2.** Meta-Analysis References

1. Bauer J, Haack A, Winning K, Wirth R, Fischer B, Uter W, Erdmann J, Schusdziarra V, Sieber C (2009) Impaired postprandial response of active ghrelin and prolonged suppression of hunger sensation in the elderly. Journals of Gerontology Series A: Biomedical Sciences and Medical Sciences 65 (3):307-311
2. Bertoli S, Magni P, Krogh V, Ruscica M, Dozio E, Testolin G, Battezzati A (2006) Is ghrelin a signal of decreased fat-free mass in elderly subjects? European journal of endocrinology 155 (2):321-330
3. Berthélemy P, Bouisson M, Vellas B, Moreau J, Albarede JL, Ribet A (1992) Postprandial cholecystokinin secretion in elderly with protein‐energy undernutrition. Journal of the American Geriatrics Society 40 (4):365-369
4. De La Maza M, Bravo A, Leiva L, Gattas V, Petermann M, Garrido F, Bunout D, Hirsch S, Barrera G, Fernandez M (2007) Fluorescent serum and urinary advanced glycoxidation end-products in non-diabetic subjects. Biological research 40 (2):203-212
5. Di Francesco V, Zamboni M, Dioli A, Zoico E, Mazzali G, Omizzolo F, Bissoli L, Solerte SB, Benini L, Bosello O (2005) Delayed postprandial gastric emptying and impaired gallbladder contraction together with elevated cholecystokinin and peptide YY serum levels sustain satiety and inhibit hunger in healthy elderly persons. The Journals of Gerontology Series A: Biological Sciences and Medical Sciences 60 (12):1581-1585
6. Di Francesco V, Zamboni M, Zoico E, Mazzali G, Dioli A, Omizzolo F, Bissoli L, Fantin F, Rizzotti P, Solerte SB (2006) Unbalanced serum leptin and ghrelin dynamics prolong postprandial satiety and inhibit hunger in healthy elderly: another reason for the “anorexia of aging”–. The American journal of clinical nutrition 83 (5):1149-1152
7. Di Francesco V, Fantin F, Residori L, Bissoli L, Micciolo R, Zivelonghi A, Zoico E, Omizzolo F, Bosello O, Zamboni M (2008) Effect of age on the dynamics of acylated ghrelin in fasting conditions and in response to a meal. Journal of the American Geriatrics Society 56 (7):1369-1370
8. Di Francesco V, Barazzoni R, Bissoli L, Fantin F, Rizzotti P, Residori L, Antonioli A, Graziani MS, Zanetti M, Bosello O (2010) The quantity of meal fat influences the profile of postprandial hormones as well as hunger sensation in healthy elderly people. Journal of the American Medical Directors Association 11 (3):188-193
9. Flint A, Bradwejn J, Vaccarino F, Gutkowska J, Palmour R, Koszycki D (2002) Aging and panicogenic response to cholecystokinin tetrapeptide: an examination of the cholecystokinin system. Neuropsychopharmacology 27 (4):663
10. Franceschini R, Corsini G, Cataldi A, Fiorucci A, Tenerelli P, Rolandi E, Barreca T (1999) Twenty-four—hour variation in serum leptin in the elderly. Metabolism-Clinical and Experimental 48 (8):1011-1014
11. Giezenaar C, Hutchison A, Luscombe-Marsh N, Chapman I, Horowitz M, Soenen S (2017) Effect of age on blood glucose and plasma insulin, glucagon, ghrelin, CCK, GIP, and GLP-1 responses to whey protein ingestion. Nutrients 10 (1):2
12. Giezenaar C, Luscombe-Marsh ND, Hutchison AT, Standfield S, Feinle-Bisset C, Horowitz M, Chapman I, Soenen S (2018) Dose-Dependent Effects of Randomized Intraduodenal Whey-Protein Loads on Glucose, Gut Hormone, and Amino Acid Concentrations in Healthy Older and Younger Men. Nutrients 10 (1):78
13. Groen BB, Horstman AM, Hamer HM, de Haan M, van Kranenburg J, Bierau J, Poeze M, Wodzig WK, Rasmussen BB, van Loon LJ (2016) Increasing insulin availability does not augment postprandial muscle protein synthesis rates in healthy young and older men. The Journal of Clinical Endocrinology & Metabolism 101 (11):3978-3988
14. Khalil T, Walker J, Wiener I, Fagan C, Townsend JC, Greeley JG, Thompson J (1985) Effect of aging on gallbladder contraction and release of cholecystokinin-33 in humans. Surgery 98 (3):423-429
15. MacIntosh CG, Andrews JM, Jones KL, Wishart JM, Morris HA, Jansen JB, Morley JE, Horowitz M, Chapman IM (1999) Effects of age on concentrations of plasma cholecystokinin, glucagon-like peptide 1, and peptide YY and their relation to appetite and pyloric motility. The American journal of clinical nutrition 69 (5):999-1006
16. MacIntosh CG, Morley JE, Wishart J, Morris H, Jansen JB, Horowitz M, Chapman IM (2001) Effect of exogenous cholecystokinin (CCK)-8 on food intake and plasma CCK, leptin, and insulin concentrations in older and young adults: evidence for increased CCK activity as a cause of the anorexia of aging. The Journal of Clinical Endocrinology & Metabolism 86 (12):5830-5837
17. MacIntosh CG, Horowitz M, Verhagen MA, Smout AJ, Wishart J, Morris H, Goble E, Morley JE, Chapman IM (2001a) Effect of small intestinal nutrient infusion on appetite, gastrointestinal hormone release, and gastric myoelectrical activity in young and older men. The American journal of gastroenterology 96 (4):997
18. Melanson KJ, Greenberg AS, Ludwig DS, Saltzman E, Dallal GE, Roberts SB (1998) Blood glucose and hormonal responses to small and large meals in healthy young and older women. The Journals of Gerontology Series A: Biological Sciences and Medical Sciences 53 (4):B299-B305
19. Moller N, O’Brien P, Nair KS (1998) Disruption of the relationship between fat content and leptin levels with aging in humans. The Journal of Clinical Endocrinology & Metabolism 83 (3):931-934
20. Moss C (2012) An investigation into age-associated undernutrition. Imperial College London, London
21. Nass R, Farhy LS, Liu J, Pezzoli SS, Johnson ML, Gaylinn BD, Thorner MO (2014) Age-dependent decline in acyl-ghrelin concentrations and reduced association of acyl-ghrelin and growth hormone in healthy older adults. The Journal of Clinical Endocrinology & Metabolism 99 (2):602-608
22. Ostlund Jr R, Yang J, Klein S, Gingerich R (1996) Relation between plasma leptin concentration and body fat, gender, diet, age, and metabolic covariates. The Journal of Clinical Endocrinology & Metabolism 81 (11):3909-3913
23. Rigamonti A, Pincelli A, Corra B, Viarengo R, Bonomo S, Galimberti D, Scacchi M, Scarpini E, Cavagnini F, Muller E (2002) Plasma ghrelin concentrations in elderly subjects: comparison with anorexic and obese patients. Journal of Endocrinology 175 (1):R1-R5
24. Rzepka E, Adamczak M, Kokot F, Wiecek A (2002) Influence of aging on plasma leptin concentration. Polskie Archiwum Medycyny Wewnetrznej 107 (2):125-133
25. Santiago J, Hallschmid M (2017) Central nervous insulin administration before nocturnal sleep decreases breakfast intake in healthy young and elderly subjects. Frontiers in neuroscience 11 (FEB) (no pagination). doi:10.3389/fnins.2017.00054
26. Sawaya AL, Fuss PJ, Dallal GE, Tsay R, McCrory MA, Young V, Roberts SB (2001) Meal palatability, substrate oxidation and blood glucose in young and older men. Physiology & behavior 72 (1-2):5-12
27. Schneider SM, Al-Jaouni R, Caruba C, Giudicelli J, Arab K, Suavet F, Ferrari P, Mothe-Satney I, Van Obberghen E, Hébuterne X (2008) Effects of age, malnutrition and refeeding on the expression and secretion of ghrelin. Clinical Nutrition 27 (5):724-731
28. Sturm K, Parker B, Wishart J, Feinle-Bisset C, Jones KL, Chapman I, Horowitz M (2004) Energy intake and appetite are related to antral area in healthy young and older subjects. The American journal of clinical nutrition 80 (3):656-667
29. Toth MJ, Arcerio PJ, Gardner AW, Calles-Escandon J, Poehlam ET (1996) Rates of free fatty acid appearance and fat oxidation in healthy younger and older men. J Appl Physiol (1985) 80 (2): 506-511 35.
30. Trahair LG, Horowitz M, Rayner CK, Gentilcore D, Lange K, Wishart JM, Jones KL (2012) Comparative effects of variations in duodenal glucose load on glycemic, insulinemic, and incretin responses in healthy young and older subjects. The Journal of Clinical Endocrinology & Metabolism 97 (3):844-851
31. Winkels RM, Jolink-Stoppelenburg A, de Graaf K, Siebelink E, Mars M, de Groot L (2011) Energy intake compensation after 3 weeks of restricted energy intake in young and elderly men. Journal of the American Medical Directors Association 12 (4):277-286
32. Woolf K, Reese CE, Mason MP, Beaird LC, Tudor-Locke C, Vaughan LA (2008) Physical activity is associated with risk factors for chronic disease across adult women's life cycle. Journal of the American Dietetic Association 108 (6):948-959
33. Yukawa M, Cummings DE, Matthys CC, Callahan HS, Frayo RS, Spiekerman CF, Weigle DS (2006) Effect of aging on the response of ghrelin to acute weight loss. Journal of the American Geriatrics Society 54 (4):648-653
34. Yukawa M, Phelan E, Callahan H, Spiekerman C, Abrass I, Weigle D (2008) Leptin levels recover normally in healthy older adults after acute diet-induced weight loss. The journal of nutrition, health & aging 12 (9):652
35. Zambrano N, Quintero J, Falque L, Souki A, Arias N, Pinero M (1996) Food intake and biochemical variables: overall nutrition and metabolic status of a group of non-institutionalized elderly in Venezuela. Archivos latinoamericanos de nutricion 46 (3):196-202
