# Supplementary material for: Differences in circulating appetite-related hormone concentrations between younger and older adults: a systematic review and meta-analysis
Source: Aging Clin Exp Res. 2019 Aug 20;32(7):1233–44. doi: 10.1007/s40520-019-01292-6 (PMC7316693; doi:10.1007/s40520-019-01292-6)
Supplement: Supplementary file 5 — Supplementary material 5 (DOCX 57 kb) [file 40520_2019_1292_MOESM5_ESM.docx]

**Article Title:** Differences in circulating appetite-related hormone concentrations between older and younger adults: a systematic review and meta-analysis

**Journal:** Aging Clinical and Experimental Research

**Author Names:** Kelsie Olivia Johnson, Oliver Michael Shannon, Jamie Matu, Adrian Holliday, Theocharis Ispoglou, Kevin Deighton

**Corresponding Author:** Dr Kevin Deighton, Institute for Sport, Physical Activity and Leisure, Leeds Beckett University, Leeds, LS6 3QS, United Kingdom (email: K.Deighton@leedsbeckett.ac.uk)

**Supplementary Table 1.** Differences in fasted hormone concentrations between older and younger adults

| Study Name | n | | Variable Assessed | | | | | | | |
| --- | --- | --- | --- | --- | --- | --- | --- | --- | --- | --- |
|  | Young | Old | Insulin | Leptin | GLP-1 | CCK | PYY | GIP | Total Ghrelin | Acylated Ghrelin |
|  |  |  |  |  |  |  |  |  |  |  |
|  |  |  |  |  |  |  |  |  |  |  |
| Bauer et al. 2010 | 8 | 8 | - | - | - | - | - | - | - | - |
| Berthélemy et al. 1992 | 32 | 19 | - | - | - | CCK8  Y 1.00±0.53  O: 0.90±0.80  (pM) | - | - | - | - |
| Bertoli et al. 2006 | 10 | 26 | Y: 3.0±1.5  O: 6.3±3.6  (μU/mL) | Y: 5380 ± 2660  O: 14200 ± 8420  (pg/ml) | - | - | - | - | Y: 2386±582.00  O: 2410.00±1272.00  (pg/ml) | - |
| De La Maza et al. 2007 | 31 | 10 | Y: 7.7±5  O: 5.4±4  ( μU/mL) | Y: 8.50 ± 7.0  O: 6.20 ± 4.0  (pg/ml) | - | - | - | - | - | - |
| Di Francesco et al. 2005 | 11 | 11 | - | - | - | Y: 12.60±13.80  O: 21.5±29.09  (pmol/L) | Y: 24.8±14.70  O: 24.9±15.18  (pmol/L) | - | - | - |
| Di Francesco et al. 2006 | 21 | 18 | Y: 3.53±21.50  O: 6.87±26.19  (mU/L) | Y: 1240 ± 3082.99  O: 4860 ± 11794.5  (pg/ml) | - | - | - | - | Y: 22.70±41.30  O: 17.80±41.01  (pg/ml) | - |
| Di Francesco et al. 2008 | 12 | 12 | - | - | - | - | - | - | - | Y: 62.80±20.40  O: 42.50±31.51  (pg/ml) |
| Di Francesco et al. 2010 | 13 | 13 | Y 20% Fat: 5.81±14.91  Y 40% Fat: 5.81±19.44  O 20% Fat: 9.36±22.03  O 40% Fat: 7.35±17.50  (U/dl) | - | Y 20% Fat: 41.20±38.04  Y 40% Fat: 38.2±24.63  O 20% Fat: 45.60±35.64  O 40% Fat: 47.90±29.81  (pg/ml) | - | - | - | - | - |
| Flint et al. 2002 | 7 | 8 | - | - | - | Total Plasma  CCK  Y: 5.69±8.93  O: 24.58±50.66  CCK-4  Y: 0.80±1.40  O: 1.50±2.70  CCK-8s  Y: 6.40±8.00  O: ±29.40±6.010  CCK-8ns  Y: 0.70±1.20  O: 1.50±3.30  (pg/ml) | - | - | - | - |
| Franceschini et al 1999 | 12 | 12 | - | Y: 7540±2276.84  O: 6950±3152.33  (pg/ml) | - | - | - | - | - | - |
|  |  |  |  |  |  |  |  |  |  |  |
| Giezenaar et al. 2018a | 31 | 10 | Y: 2.60±1.95  O: 2.09±0.80  (mU/L) | - | Y: 25.53±7.54  O: 23.12±9.07  (pmol/L) | - | Y: 34.98±13.02  O: 26.10±5.55  (pmol/L) | Y : 12.12±1.96  O: 14.78±5.06 | Y: 1480.00 ± 1207.70  O: 1505.00 ±296.09  (pg/ml) | - |
| Giezenaar et al. 2018b | 15 | 19 | Y 120kcal: 5.66±2.72  Y 280kcal: 4.39±3.64  O 120kcal: 4.78±2.20  Y 280kcal: 4.78±2.20  (mU/L) | - | Y 120kcal: 22.40±4.40  Y 280kcal: 21.10±9.60  O 120kcal: 29.40±9.60  Y 280kcal: 34.50±18.00  (pmol/L) | Y 120kcal: 3.42±2.84  Y 280 kcal: 3.13±1.68  O 120 kcal: 4.78±2.20  O 280 kcal: 4.78±2.20  (pmol/L) | - | Y 120kcal: 15.90±4.80  Y 280kcal: 14.70±9.60  O 120kcal: 12.2 ± 9.20  O 280kcal: 12.2 ± 9.2  (pmol/L) | Y 120kcal: 1420.00 ± 320.00  Y 280kcal: 1590.00 ± 920.00  O 120kcal: 1420.00 ± 760.00  O 280kcal: 1470.00 ± 640.00  (pg/ml) | - |
| Groen et al. 2016 | 18 | 6 | Y: 9.08±26.41  O: 10.41±17.05  (mU.L^-1^) | - | - | - | - | - | - | - |
| Moss. 2012 | 12 | 12 | Y: 9.87±12.13  O: 27.3 ± 17.39  (pmol/L) | - | Y:217.00±203.65  O: 171±144.52  (pmol/L) | - | Y: 31.00±36.06  O: 26.70±67.12  (pmol/L) | - | Y: 2153.43 ± 2301.93  O: 3161.06 ± 2946.96  (pg/ml) | Y: 980.67±1215.30  O: 808.80±982.32  (pg/ml) |
| Khalil et al. 1985 | 10 | 10 | - | - | - | Y: 120.00±56.12  O: 194.00±67.39  (pg/ml) | - | - | - | - |
| MacIntosh et al. 1999 | 10 | 26 | - | - | Y Glucose: 6.26±6.09  Y Lipid: 7.68±9.21  O Glucose: 8.56±11.09  O Lipid:10.20±10.84  (pmol/L) | Y Glucose: 6.05±8.70  Y Lipid: 3.07±1.48  O Glucose: 8.48±12.84  O Lipid: 4.48±2.09  (pmol/L) | Y Glucose: 9.99±5.58  Y Lipid: 10.90±5.23  O Glucose: 10.00±6.90  O Lipid:11.90 ±6.22  (pmol/L) | - | - | - |
| MacIntosh et al. 2001a | 21 | 19 | Y: 4.71±33.24  O: 7.19±41.46  (mU/L) | - | Y Glucose: 11.30±22.71  Y Lipid: 13.10±20.55  O Glucose: 9.44±20.37  O Lipid: 10.50±12.98  (pmol/L) | - | - | Y Glucose 22.90 ± 40.38  Y Lipid: 28.00 ± 31.37  O Glucose: 25.00 ± 62.38  O Lipid: 25.20 ± 33.53  (pmol/L) | - | - |
| MacIntosh et al. 2001b | 10 | 9 | Y Control: 7.27±25.63  Y Low Dose: 7.44±26.36  Y High Dose: 5.92±25.11  O Control: 5.15±23.94  O Low Dose: 5.67±25.11  O High Dose: 4.63±20.33  ( mU/L) | Control  Y:6330±8037  O: 9746±8857  Glucose  Y:7003±8021  O: 8360±8972  Lipid  Y: 7110±8021  O: 9650±12263  (pg/ml) |  | Plasma CCK  Y Control: 2.31±2.46  Y Low Dose: 2.19±2.08  Y High Dose: 2.28±2.08  O Control:  3.79±4.43  O Low Dose: 3.69±6.30  O High Dose: 3.79±3.95  Plasma CCK-8 receptor  Y Control: 1.27±16.28  Y Low Dose: 1.80±18.76  Y High Dose: 1.36±17.84  O Control: 3.80±16.63  O Low Dose: 7.33±27.26  O High Dose: 4.08±15.10  (pmol/L) |  |  |  |  |
|  |  |  |  |  |  |  |  |  |  |  |
| Melanson et al. 1998 | 12 | 7 | Y: 14.12±28  O: 17.16±25.53  (μU/mL) |  |  |  |  |  |  |  |
| Moller et al. 1998 | 7 | 7 | Y: 6±3.2  O:6.9±3.7  (μU/mL) | Y:8100 ± 7100  O: 11200±10000  (pg/ml) | - | - | - | - | - | - |
| Nass et al. 2014 | 18 | 30 | - | - | - | - | - | - | - | - |
| Ostlund et al. 1996 | 89 | 115 | - | Y: 17800.00±16037.80  O: 8360.00±6434.28  (pg/ml) | - | - | - | - | - | - |
| Rigamonti et al. 2002 | 10 | 10 | Y: 3.5±2.08  O: 10.5±6.88  (μU/mL) | - | - | - | - | - | - | - |
| Rzepka et al. 2002 | 30 | 28 | - | Y: 10633 13055  O:12633±12670  (pg/ml) | - | - | - | - | - | - |
| Santigo et al. 2017 | 8 | 8 | - | - | - | - | - | - | - | - |
| Sawaya et al. 2001 | 8 | 6 | Y Low Palatability: 12.1±23.71  Y High Palatability: 12.4±18.34  O Low Palatability: 11.9±9  O High Palatability:  11.7±9.6  (μU/mL) | - | - | - | - | - | - | - |
| Schneider et al. 2008 | 13 | 13 | - | - | - | - | - | - | Y: 948.00 ± 334.73  O: 1215.00 ± 453.10  (pg/ml) | - |
|  |  |  |  |  |  |  |  |  |  |  |
| Sturm et al. 2004 | 12 | 12 | Y Control: 12.03±20.21  Y Low Dose: 11.4±18.71  Y High Dose: 13.1±20.44  O Control: 8.38±23.39  O Low Dose: 8.99±22.52  O High Dose: 8.68±25.98  (mmol/L) | - | - | Y Control: 2.23±5.37  Y Low Dose: 2.28±6.24  Y High Dose: 1.75±5.20  O Control: 4.69±9.05  O Low Dose: 4.73±10.05  O High Dose: 4.67±7.97  (pmol/L) | - | - | - | - |
| Toth et al. 1996 | 10 | 9 | Y: 49±29.70  O: 65 ± 32.86  (pmol/L) | - | - | - | - | - | - | - |
| Trahair et al. 2012 | 14 | 15 | Y Control: 3.6±1.90  Y 1kcal/min: 3.7±1.73  Y 2kcal/min: 3.3±1.04  Y 3kcal/min: 3.6±1.39  O Control: 7.3±2.77  O 1kcal/min: 6.9±3.81  O 2kcal/min: 6.7±3.11  O 3kcal/min: 6.1±2.08  (mU/L) | - | Y Control: 17.40±11.43  Y 1kcal/min: 19.4±6.93  Y 2kcal/min: 18.9±5.20  Y 3kcal/min: 21.30±7.27  O Control: 14.28±6.18  O 1kcal/min: 14.50±4.85  O 2kcal/min: 15.80±5.89  O 3kcal/min: 16.00±6.24  (pmol/L) | - | - | Y Control: 10.2±4.85  Y 1kcal/min: 11.5±5.54  Y 2kcal/min: 10.2±5.54  Y 3kcal/min: 10±4.85  O:12.4±6.93  O 1kcal/min: 13.5±8.66  O 2kcal/min: 13.1±7.62  O 3kcal/min: 12.2±5.20  (pmol/L) | - | - |
| Winkels et al. 2011 | 10 | 12 |  |  |  | CCK8  Y: 0.38±1.11  O: 0.43±1.34  (pmol/L) |  |  |  |  |
| Woolf et al. 2008 | 49 | 47 | Y: 18±6  O: 19±7  (μU/mL) | Y: 12000±600  O: 19000±1000  (pg/ml) | - | - | - | - | - | - |
| Yukawa et al. 2006 | 57 | 50 |  | - |  |  |  |  |  |  |
| Yukawa et al. 2008 | 10 | 10 | - | - | - | - | - | - | - | - |
| Zambrano et al. 1996 | 16 | 16 | Y: 11.38±6.01  O: 10.89±6.37  (μU/mL) | - |  |  |  |  |  |  |

**Supplementary Table 2.** Differences in postprandial hormone concentrations between older and younger adults

| Study Name | N | | Variable Assessed | | | | | | | |
| --- | --- | --- | --- | --- | --- | --- | --- | --- | --- | --- |
|  | Young | Old | Insulin | Leptin | GLP-1 | CCK | PYY | GIP | Total Ghrelin | Acylated Ghrelin |
|  |  |  |  |  |  |  |  |  |  |  |
|  |  |  |  |  |  |  |  |  |  |  |
| Bauer et al. 2010 | 8 | 8 | Y: 9.31±7.12  O: 16.49±11.99  (μg/ml) | Y: 12±10.59  O: 14.2±8.53  (pg/ml) | - | - | - | - | Y: 852.75±271.10  O: 913.25±427.82  (pg/ml) | Y: 72.33±45.04  O: 101.48±62.18  (pg/ml) |
| Berthélemy et al. 1992 | 32 | 19 | - | - | - | CCK8  Y: 2.06±2.34  O: 1.9±1.90  (pM) | - | - | - | - |
| Bertoli et al. 2006 | 9 | 10 | Y: 20.5±7.1  O: 46.3±33.7  (μU/mL) | Y: 4880±2290  O: 13040±7950  (pg/ml) | - | - | - | - | Y: 2080.00±602.00  O: 1888.00± 764.00  (pg/ml) | - |
| De La Maza et al. 2007 | 23 | 24 | - | - | - | - | - | - | - | - |
| Di Francesco et al. 2005 | 11 | 11 | - | - | - | Y: 12.34±24.81  O: 30.04±35.82  (pmol/L) | Y: 24.5±23.89  O: 31.14±15.07  (pmol/L) | - | - | - |
| Di Francesco et al. 2006 | 21 | 18 | Y: 20.37±22.39  O: 41.34±61.96  (mU/L) | Y: 1020.00±2836.2  O: 4780.00±6262.58 | - | - | - | - | Y: 18.52±38.35  O: 18.42±30.36  (pg/ml) | - |
| Di Francesco et al. 2008 | 12 | 12 | - | - | - | - | - | - | - | Y: 59.68±76.30  O: 39.88±30.91  (pg/ml) |
| Di Francesco et al. 2010 | 13 | 13 | Y 20% Fat: 25±57.84  Y 40% Fat: 8.4±45.43  O 20% Fat: 3.55±42.90  O 40% Fat: 33.2±58.37  (U/dl) | - | Y 20% Fat: 43.70±45.20  Y 40% Fat: 42.86±43.57  O 20% Fat: 41.57±39.23  O 40% Fat: 51.50±32.80  (pg/ml) |  | - | - | - | - |
| Flint et al. 2002 | 7 | 8 | - | - | - | - | - | - | - | - |
| Franceschini et al 1999 | 12 | 12 | Y: 8150±2687.936  O: 7820.00±3498.74  (pg/ml) | - | - | - | - | - | - | - |
|  |  |  |  |  |  |  |  |  |  |  |
| Giezenaar et al. 2018a | 31 | 10 | Y 30 kcal: 6.60±8.99  Y 90kcal: 14.33±9.05  Y 180kcal: 24.28±12.55  O 30kcal: 4.07±2.51  O 90 kcal: 11.30±7.21  O 180kcal: 18.05±12.72  (mU/L) | - | Y 30kcal: 29.28±9.78  Y 90kcal:35.63±10.53  Y 180kcal: 39.88±13.93  O 30kcal: 23.90±7.33  O 90kcal: 27.08±11.43  O 180kcal: 35.40±15.37  (pmol/L) | - | Y 30kcal: 35.88±7.31  Y 90kcal: 37.18±13.28  Y 180kcal: 42.00±18.91  O 30kcal: 26.18±5.87  O 90 kcal: 29.35±6.33  O 180kcal: 36.45±22.30  (pmol/L) | Y 30kcal: 20.9±4.51  Y 90kcal: 24.6±7.28  Y 180kcal: 32.2±7.43  O 30kcal: 26.6±9.02  O 90kcal: 38.83±11.99  O 180kcal: 45.78±16.75  (pmol/L) | Y 30kcal: 1456.25±577.81  Y 90kcal: 1247.50±393.56  Y 180kcal: 1180.00±681.86  O 30kcal: 1485.00±477.14  O 90kcal:  1387.50±660.20  180kcal: 1350.00±439.03  (pg/ml) | - |
| Giezenaar et al. 2018b | 15 | 19 | Y 120kcal: 11.44±7.86  Y 280kcal: 15.48±9.38  O 120kcal: 1.49±11.59  O 280kcal: 5.87±12.17  (mU/L) | - | Y 120kcal: 27.59±9.68  Y 280kcal: 32.69±10.93  O 120kcal: 33.90±12.45  O 280kcal: 41.33±17.01  (pmol/L) | Y 120kcal: 4.72±1.42  Y 280 kcal: 5.67±2.32  O 120kcal: 7.04±3.32  O 280 kcal: 8.27±3.30  (pmol/L) | - | Y 120kcal: 25.48±8.89  Y 280kcal: 27.88±10  .22  O 120kcal: 27.12±12.72  O 280kcal: 32.10±14.86  (pmol/L) | Y 120kcal: 1375.56±966.81  Y 280kcal: 1247.78±804.53  O 120kcal: 1415.56±687.29  O 280kcal: 1350.00±653.58  (pg/ml) | - |
| Groen et al. 2016 | 18 | 6 | Y: 10.32±23.51  O: 10.76±19.38  (mU.L^-1^) | - | - | - | - | - | - | - |
| Moss. 2012 | 12 | 12 | Y: 39.52±76.07  O: 61.34±39.82  (pmol/L) | - | Y: 302.40±150.13  O: 295.20±386.41  (pmol/L) | - | Y: 44.32±50.64  O: 54.22±35  (pmol/L) | - | Y: 1485.50±2093.71  O:2406.18 ±1831.15  (pg/ml) | Y: 448.82±877.86  O: 359.71±441.30  (pg/ml) |
| Khalil et al. 1985 | 10 | 10 | - | - | - | Y: 196.00±100.03  O: 273.67 ± 85.14  (pg/ml) | - | - | - | - |
| MacIntosh et al. 1999 | 10 | 26 | - | - | Y Glucose: 19.88±21.12  Y Lipid: 28.72±22.25  O Glucose: 23.98±29.84  O Lipid:32.73±28.05  (pmol/L) | Y Glucose: 19.74 ± 21.06  Y Lipid: 8.39 ± 5.21  O Glucose: 23.78±28.51  O Lipid: 11.21±6.15  (pmol/L) | Y Glucose: 14.54 ± 8.55  Y Lipid: 31.27 ± 25.50  O Glucose: 14.68±10.33  O Lipid: 28.89±16.99  (pmol/L) | - | - | - |
| MacIntosh et al. 2001a | 21 | 19 | Y: 54.12±78.61  O: 98.07±764.80  (mU/L) | - | Y Glucose: 23.20±23.47  Y Lipid: 26.98±34.93  O Glucose: 25.68±32.46  O Lipid: 32.63±44.16  (pmol/L) | - | - | Y Glucose: 95.77±97.38  Y Lipid: 26.98±34.93  O Glucose: 83.85±109.46  O Lipid: 78.88±76.63  (pmol/L) | - |  |
| MacIntosh et al. 2001b | 10 | 9 | Y Control: 65.54±59.53  Y Low Dose: 13.4±26.36  Y High Dose: 11.7±19.99  O Control: 46.99±42.50  O Low Dose: 8.56±25.84  O High Dose: 11.2±25.29  (mU/L) | Y Control: 6237.50±8213.25  Y Low dose: 5620±6304.66  Y High Dose: 6080.00±7101.41  O Control: 8360.00±8972.02  O Low Dose: 8390.00±11466.20  O High Dose: 9320.00±14445.33 (pg/ml) | - | Plasma CCK:  Y Control:2.91±1.70  Y Low Dose: 2.69±1.63  Y High Dose: 2.92±1.39  O Control: 4.25±2.48  O Low Dose: 4.73±1.97  O High Dose: 4.76±2.70  Plasma CCK-8  Y Control: 3.98±19.29  Y Low Dose: 3.84±16.80  Y High Dose: 3.79±16.84  O Control: 10.54±20.12  O Low Dose: 11.90±28.02  O High Dose: 8.83±18.64  (pmol/L) | - | - | - | - |
|  |  |  |  |  |  |  |  |  |  |  |
| Melanson et al. 1998 | 12 | 7 | Y 1046 kJ :27.48±29.51  Y 2092 kJ: 33.74±38.51  Y 4184 kJ:  48.4±63.63  O 1046 kJ: 27.86±34.53  O 2092 kJ: 47.58±66.49  O 4184 kJ: 43.34±68.30  (μU/mL) | - | - | - | - | - | - | - |
| Moller et al. 1998 | 7 | 7 | - | - | - | - | - | - | - | - |
| Nass et al. 2014 | 18 | 30 | Y: 9.8±2.82  O: 13.1±9.06  (μIU/mL) | - | - | - | - | - | - | Y: 27.80±11.03  O: 14.70±5.14  (pg/ml) |
| Ostlund et al. 1996 | 89 | 115 | - | - | - | - | - | - | - | - |
| Rigamonti et al. 2002 | 10 | 10 | - | - | - | - | - | - | - | - |
| Rzepka et al. 2002 | 12 | 12 | - | - | - | - | - | - | - | - |
| Santigo et al. 2017 | 8 | 8 | Y: 41.81±17.48  O: 52.42±9.65  (pmol/L) | - | - | - | - | - | - | - |
| Sawaya et al. 2001 | 8 | 6 | Y Low Palatability: 40.10±36.28  Y High Palatability: 45.20±40.98  O Low Palatability: 49.51±46.90  O High Palatability: 51.49±54.79  (μU/mL) | - | - | - | - | - | - | - |
| Schneider et al. 2008 |  |  | Y: 15.24±10.54  O: 17.74±10.06  (u/l) | Y: 7800±5900  O: 18800±11500  (pg/ml) |  |  |  |  | Y: 921.00±347.59  O: 1108.56±451.95  (pg/ml) | Y: 51.40±32.50  O: 44.70±34.60  (ng/ml) |
|  |  |  |  |  |  |  |  |  |  |  |
| Sturm et al. 2004 | 12 | 12 | Y Low Dose: 42.48±82.92  Y High Dose: 53.76±65.04  O Low Dose: 39.84±52.73  O High Dose: 52.42±49.59  (mmol/L) | - | - | Y Low Dose: 6.52±7.48  Y High Dose: 9.15±11.47  O Low Dose: 14.10±22.21  O High Dose: 16.12±22.44  (mmol/L) | - | - | - | - |
| Toth et al. 1996 | 10 | 9 | - | - | - | - | - | - | - | - |
| Trahair et al. 2012 | 14 | 15 | Y 1kcal/min: 8.21±4.60  Y 2kcal/min: 24.63±28.45  Y 3kcal.min: 46.45±46.44  (mU/L)  O 1kcal/min: 13.34±5.149  O 2kcal/min: 33.53±34.00  O 3kcal/min: 71.35±65.62  (mU/L) | - | Y 1kcal/min: 19.31±17.71  Y 2kcal/min: 27.44±11.28  Y 3kcal/min: 33.38±11.05  O 1kcal/min: 24.16±11.36  O 2kcal/min: 32.54±13.83  O 3kcal/min: 40.44±15.42  (pmol/L) | - | - | Y: 1kcal/min: 14.43±4.38  2kcal/min: 21.06±12.82  3kcal/min: 35.5±20.34  O 1kcal/min: 13.86±3.83  O 2kcal/min: 17.23±11.83  O 3kcal/min: 35.93±18.46  (pmol/L) | - | - |
| Schneider et al. 2008 | 13 | 13 | - | - | - | - | - | - | - | - |
| Winkels et al. 2011 | 10 | 12 | - | - | - | CCK8  Y: 0.87±2.11  O: 0.81±0.87  (pmol/L) | - | - | - | - |
| Woolf et al. 2008 | 20 | 20 | - | - | - | - | - | - | - | - |
| Yukawa et al. 2006 | 57 | 50 | - | - | - | - | - | - | Y: 329.58±63.01  O: 420.83±335.87  (pg/ml) | - |
| Yukawa et al. 2008 | 10 | 10 | - | Y: 23160±5790  O: 30250±6290  (pg/ml) | - | - | - | - | - | - |
| Zambrano et al. 1996 | 16 | 16 | - | - | - | - | - | - | - | - |

**Supplementary Table 3.** Differences in energy intake between older and younger adults

| Study Name | Energy Intake (kJ) |
| --- | --- |
|  |  |
| Zambrano et al. 1996 | Y: 7808.81 ± 3143.93 O: 7413.58 ± 59 |
| Santigo et al. 2017 | Y: 4722.23 ± 296.14 O: 5095.65 ± 532.96 |
| MacIntosh et al. 2001b | Y: 2690 ± 305 O: 4000 ± 440 |
| MacIntosh et al. 2001a | Y: 9590 ± 824 O: 8681 ± 469 |
| MacIntosh et al. 1999 | Y: 10209 ± 623 O: 7381 ± 950 |
| Sturm et al. 2004 | Control Y: 5330.42 ± 1478 ± 37 Low Dose Y: 4393.2 ± 950.30 High Dose Y: 3476.90 ± 1492.86 Control O: 4506.17 ± 173.75 Low Dose Control O: 3953.88 ± 1666.79 High Dose |
| Moss 2012 | Y: 4263.08 ± 1474.02 O: 1738.95 ± 491.20 |
| Woolf et al. 2008 | Y: 7681.82 ± 1443.48 O: 6585.62 ± 1430.93 |
| Toth et al. 1996 | Y: 12384.64 ± 652.70 O:9070.91 ± 372.38 |
| Arciero et al. 2009 | Y: 9849.14 ± 3530.04 O: 8452.94 ± 1904.56 |
| Giezenaar et al. 2018a | Y Control: 5313.68 ± 627.6 Y 30kcal: 4698.63 ± 631.84 Y 90 kcal: 4301.15 ± 681.99 Y 180kcal: 3560.58 ± 673.62  O Control: 4468.51 ± 389.11 O 30kcal: 4723.74 ± 380.74 90kcal: 4698.63 ± 410.03 O 180kcal: 3761.42 ± 430.95 |
| Giezenaar et al. 2018b | Y 120kcal: 4029 ± 331.00 Y 280kcal: 3966.00 ± 343.00  O 120kcal: 3360.00 ± 314.00 O 280kcal: 3318.00 ± 326 |

**Supplementary Table 4**. Difference in fasted and postprandial hunger between older and younger adults

| Study Name | n | |  |  |
| --- | --- | --- | --- | --- |
|  | Young | Old | Fasting | Postprandial |
| Bauer et al. 2010 | 8 | 8 | - | Y: 4.93±2.37  O: 4.20±2.99  (cm) |
| Berthélemy et al. 1992 | 32 | 19 | - | - |
| Bertoli et al. 2006 | 10 | 26 | - | - |
| De La Maza et al. 2007 | 31 | 10 | - | - |
| Di Francesco et al. 2005 | 11 | 11 | Y: 7.34±3.57  O: 4.81±7.37  (cm) | Y : 4.07±43.33  O: 1.88±7.11  (cm) |
| Di Francesco et al. 2006 | 21 | 18 | Y: 7.30±1.13  O: 4.80±3.11  (cm) | Y: 4.06±2.45  O: 1.88±1.74  (cm) |
| Di Francesco et al. 2008 | 12 | 12 | - | - |
| Di Francesco et al. 2010 | 13 | 13 | - | - |
| Flint et al. 2002 | 7 | 8 | - | - |
| Franceschini et al 1999 | 12 | 12 | - | - |
|  |  |  |  |  |
| Giezenaar et al. 2018a | 31 | 10 | Y: 67.00±15.81  O: 45.00±28.46  (mm) | - |
| Giezenaar et al. 2018b | 15 | 19 | - | - |
| Groen et al. 2016 | 18 | 6 | - | - |
| Moss. 2012 | 12 | 12 | Y: 67.53±5.04  O: 18.37±9.85  (mm) | Y: 39.08±20.62  O: 13.15±8.57  (mm) |
| Khalil et al. 1985 | 10 | 10 | - | - |
| MacIntosh et al. 1999 | 10 | 26 | Y: 5.20±2.11  O: 1.90±1.70  (cm) | - |
| MacIntosh et al. 2001a | 21 | 19 | Y Control: 6.05±12.20  Y Glucose: 5.72±3.24  Y Lipid: 5.88±21.20  O Control: 3.18±3.52  O Glucose: 3.09±3.53  O Lipid: 3.26±3.82  (cm) | Y Glucose: 5.11±3.83  Y Lipid: 6.01±3.30  O Glucose: 3.26±3.48  O Lipid: 3.71±3.72  (cm) |
| MacIntosh et al. 2001b | 10 | 9 | Y: 6.10±1.39  O: 4.00±1.03  (cm) | - |
| Melanson et al. 1998 | 12 | 7 |  | - |
| Moller et al. 1998 | 7 | 7 | - | - |
| Nass et al. 2014 | 18 | 30 | - | - |
| Ostlund et al. 1996 | 89 | 115 | - | - |
| Rigamonti et al. 2002 | 10 | 10 | - | - |
| Rzepka et al. 2002 | 30 | 28 | - | - |
| Santigo et al. 2017 | 8 | 8 | Y: 67.03±18.28  O: 46.25±18.60  (unit not stated) | - |
| Sawaya et al. 2001 | 8 | 6 | Y: 49.25±31.37  O: 68.8±32.53  (unit not stated) | - |
| Schneider et al. 2008 | 13 | 13 | Y: 37.80±37.04 31.75±34.47  (cm) | Y: 15.9±24.69  O: 29.28±31.34  (cm) |
|  |  |  |  |  |
| Sturm et al. 2004 | 12 | 12 | Y: 69.88±43.03  O: 47.17±56.19  (mm) | Y Low Dose: 51.88±42.77  Y High Dose: 51.87±42.77  O Low Dose: 31.19±50.10  O High Dose: 30.00±51.97  (mm) |
| Toth et al. 1996 | 10 | 9 | - | - |
| Trahair et al. 2012 | 14 | 15 | - | - |
| Winkels et al. 2011 | 10 | 12 | Y: 5.89±9.30  O: 4.05±2.62  (cm) | - |
| Woolf et al. 2008 | 49 | 47 | - | Y: 4.65±5.33  O: 3.95±3.02  (cm) |
| Yukawa et al. 2006 | 57 | 50 |  | - |
| Yukawa et al. 2008 | 10 | 10 | - | - |
| Zambrano et al. 1996 | 16 | 16 | - | - |
