# Supplementary material for: Differences in circulating appetite-related hormone concentrations between younger and older adults: a systematic review and meta-analysis
Source: Aging Clin Exp Res. 2019 Aug 20;32(7):1233–44. doi: 10.1007/s40520-019-01292-6 (PMC7316693; doi:10.1007/s40520-019-01292-6)
Supplement: Supplementary file 6 — Supplementary material 6 (DOCX 39 kb) [file 40520_2019_1292_MOESM6_ESM.docx]

**Article Title:** Differences in circulating appetite-related hormone concentrations between older and younger adults: a systematic review and meta-analysis

**Journal:** Aging Clinical and Experimental Research

**Author Names:** Kelsie Olivia Johnson, Oliver Michael Shannon, Jamie Matu, Adrian Holliday, Theocharis Ispoglou, Kevin Deighton

**Corresponding Author:** Dr Kevin Deighton, Institute for Sport, Physical Activity & Leisure, Leeds Beckett University, Leeds, LS6 3QS, United Kingdom (email: K.Deighton@leedsbeckett.ac.uk)

| **Supplementary Table 23.** Sensitivity analysis and Egger’s Regression for fasted appetite-related hormone concentrations and hunger | | | | | | | | | | | | | | | | | |
| --- | --- | --- | --- | --- | --- | --- | --- | --- | --- | --- | --- | --- | --- | --- | --- | --- | --- |
|  | | **Sensitivity Analysis** | | | | | | | | | | **Eggers Regression** | | | | | |
| **Variable** | | **Changes Detected?** | | **Study Author** | **SMD** | | **95% CI** | | | **p** | | **Intercept** | | **95% CI** | | **p** | |
| **Acylated ghrelin** | | N/A | | | | | | | | | | | | | | | |
| **Total ghrelin** | | Minor changes only | | - | - | | - | | | - | | 0.690 | | -2.319 to 3.700 | | 0.559 | |
| **CCK** | | Minor changes only | | - | - | | - | | | - | | 1.343 | | -0.440 to 3.126 | | 0.118 | |
| **GLP-1** | | Minor changes only | | - | - | | - | | | - | | 3.007 | | -5.887 to 11.902 | | 0.425 | |
| **Leptin** | | ↓ SMD | | (Woolf et al., 2008) | 0.38 | | -0.24 to 1.00 | | | 0.225 | |  | | 2.966 to 16.448 | | 0.011 | |
|  | | ↓ SMD | | (de la Maza et al., 2004) | 1.12 | | -0.01 to 2.24 | | | 0.051 | | 9.707 | |  |  |  | |
| **Insulin** | | ↓ SMD | |  | 0.20 | | -0.06 to 0.45 | | | 0.138 | | 1.474 | | -1.221 to 4.169 | | 0.265 | |
|  | | ↓ SMD | | (Moss. 2012) | 0.20 | | -0.06 to 0.45 | | | 0.138 | |  |  |  |  |  |  |
|  | | ↓ SMD | | (Rigamonti et al., 2002) | 0.19 | | -0.06 to 0.444 | | | 0.145 | |  |  |  |  |  |  |
|  | | ↓ SMD | | (Toth et al., 1996) | 0.22 | | -0.05 to 0.49 | | | 0.107 | |  | |  | |  | |
|  | | ↓ SMD | | (Trahair et al., 2012) | 0.13 | | -0.08 to 0.35 | | | 0.210 | |  | |  | |  | |
|  | | ↓ SMD | | (Woolf et al., 2008) | 0.22 | | -0.05 to 0.50 | | | 0.113 | |  | |  | |  | |
| **PYY** | | Minor changes only | | - | - | | - | | | - | | 6.275 | | -2.984 to 15.534 | | 0.100 | |
| **GIP** | | Minor changes only | | - | - | | - | | | - | | 6.555 | | 0.710 to 12.400 | | 0.040 | |
| **Hunger** | | Minor changes only | | - | - | | - | | | - | | -8.222 | | -10.677 to -5.767 | | <0.0005 | |
|  | |  | |  |  | |  | | |  | |  | |  | |  | |
|  | |  | |  |  | |  | | |  | |  | |  | |  | |
| **Supplementary Table 24.** Sensitivity analysis and Egger’s Regression for postprandial appetite-related hormone concentrations and hunger | | | | | | | | | | | | | | | | |  |
|  | **Sensitivity Analysis** | | | | | | | | | | **Eggers Regression** | | | | | |  |
| **Variable** | **Changes Detected?** | | **Study Author** | | | **SMD** | | **95% CI** | ***p*** | | ***Intercept*** | | ***95% CI*** | | ***p*** | |  |
| **Acylated ghrelin** | Minor changes only | | - | | | - | | - | - | | -7.760 | | -12.586 to -2.934 | | 0.014 | |  |
| **Total ghrelin** | Minor changes only | | - | | | - | | - | - | | 0.298 | | -2.198 to 2.795 | | 0.780 | |  |
| **CCK** | Minor changes only | | - | | | - | | - | - | | 0.804 | | -1.872 to 3.480 | | 0.490 | |  |
| **GLP-1** | ↑SMD | | (Giezenaar et al., 2018) | | | 0.14 | | 0.02 to 0.48 | 0.034 | | -1.299 | | -9.036 to 6.438 | | 0.342 | |  |
| **Leptin** | Minor changes only | | - | | | - | | - | - | | -2.220 | | -3.411 to 7.852 | | 0.357 | |  |
| **Insulin** | ↓ SMD | | (Bertoli et al., 2006) | | | 0.13 | | -0.02 to 0.28 | 0.102 | |  | |  | |  | |  |
|  | ↓ SMD | | (Santiago & Hallschmid, 2017) | | | 0.12 | | -0.04 to 0.27 | 0.132 | |  |  |  |  |  |  |  |
|  | ↓ SMD | | (Bauer et al., 2010) | | | 0.13 | | -0.02 to 0.28 | 0.096 | |  |  |  |  |  |  |  |
|  | ↓ SMD | | (Trahair et al., 2012) | | | 0.15 | | -0.01 to 0.32 | 0.083 | |  |  |  |  |  |  |  |
|  | ↑SMD | | (Sawaya et al., 2001) | | | 0.17 | | 0.00 to 0.34 | 0.054 | |  | |  | |  | |  |
|  | ↓ SMD | | (Di Francesco et al., 2006) | | | 0.16 | | -0.01 to 0.32 | 0.059 | | 2.106 | | -0.084 to 4.230 | | 0.058 | |  |
|  | ↓ SMD | | (Nass et al., 2008) | | | 0.16 | | -0.01 to 0.32 | 0.083 | |  | |  | |  | |  |
|  | ↑SMD | | (Schneider et al., 2008) | | | 0.16 | | 0.00 to 0.33 | 0.053 | |  | |  | |  | |  |
|  |  | |  | | |  | |  |  | |  | |  | |  | |  |
| **PYY** | ↑SMD | | (MacIntosh et al., 1999) | | | 0.43 | | 0.03 to 0.82 | 0.032 | | -1.810 | | -9.227 to 5.606 | | 0.404 | |  |
| **GIP** | Minor changes only | | - | | | - | | - | - | | 11.348 | | -53.226 to 75.923 | | 0.528 | |  |
| **Hunger** | ↓ SMD | | (MacIntosh et al., 2001) | | | -0.39 | | -0.75 to -0.03 | 0.036 | | -2.467 | | -7.194 to 2.260 | | 0.249 | |  |

| **Supplementary Table 25.** Sensitivity analysis and Egger’s Regression for energy intake | | | | | | | | |
| --- | --- | --- | --- | --- | --- | --- | --- | --- |
|  | **Sensitivity analysis** | | | | | **Egger’s Regression** | | |
|  | **Changes Detected?** | **Study Author** | **SMD** | **95% CI** | ***p*** | **Intercept** | **95% CI** | ***p*** |
| **Energy Intake** | ↑SMD | (Toth et al., 1996) | -0.52 | -1.15 to 0.12 | 0.11 | -3.357 | -9.375 to 2.660 | 0.239 |

Bauer, J. M., Haack, A., Winning, K., Wirth, R., Fischer, B., Uter, W., . . . Sieber, C. C. (2010). Impaired postprandial response of active ghrelin and prolonged suppression of hunger sensation in the elderly. *J Gerontol A Biol Sci Med Sci, 65*(3), 307-311. doi:10.1093/gerona/glp174

Bertoli, S., Magni, P., Krogh, V., Ruscica, M., Dozio, E., Testolin, G., & Battezzati, A. (2006). Is ghrelin a signal of decreased fat-free mass in elderly subjects? *Eur J Endocrinol, 155*(2), 321-330. doi:10.1530/eje.1.02220

de la Maza, M. P., Vivien, Z. G., Zavala, A. R., Cataldo, V. D., Guerra, J. S., Gladys, A. B., . . . Daniel, B. B. (2004). [Weight maintenance in humans. Could it mimic calorie restriction of animal models?]. *Rev Med Chil, 132*(10), 1166-1172.

Di Francesco, V., Zamboni, M., Zoico, E., Mazzali, G., Dioli, A., Omizzolo, F., . . . Bosello, O. (2006). Unbalanced serum leptin and ghrelin dynamics prolong postprandial satiety and inhibit hunger in healthy elderly: another reason for the "anorexia of aging". *Am J Clin Nutr, 83*(5), 1149-1152.

Giezenaar, Caroline, Luscombe-Marsh, Natalie D, Hutchison, Amy T, Standfield, Scott, Feinle-Bisset, Christine, Horowitz, Michael, . . . Soenen, Stijn. (2018). Dose-Dependent Effects of Randomized Intraduodenal Whey-Protein Loads on Glucose, Gut Hormone, and Amino Acid Concentrations in Healthy Older and Younger Men. *Nutrients, 10*(1), 78.

MacIntosh, C. G., Andrews, J. M., Jones, K. L., Wishart, J. M., Morris, H. A., Jansen, J. B., . . . Chapman, I. M. (1999). Effects of age on concentrations of plasma cholecystokinin, glucagon-like peptide 1, and peptide YY and their relation to appetite and pyloric motility. *Am J Clin Nutr, 69*(5), 999-1006.

MacIntosh, C. G., Horowitz, M., Verhagen, M. A., Smout, A. J., Wishart, J., Morris, H., . . . Chapman, I. M. (2001). Effect of small intestinal nutrient infusion on appetite, gastrointestinal hormone release, and gastric myoelectrical activity in young and older men. *Am J Gastroenterol, 96*(4), 997-1007. doi:10.1111/j.1572-0241.2001.03684.x

Nass, R, Pezzoli, Ss, Oliveri, Mc, Patrie, Jt, Harrell, Fe, Clasey, Jl, . . . Thorner, Mo. (2008). Effects of an oral ghrelin mimetic on body composition and clinical outcomes in healthy older adults: a randomized trial. *Annals of internal medicine, 149*(9), 601-611. <http://onlinelibrary.wiley.com/o/cochrane/clcentral/articles/501/CN-00651501/frame.html>

Rigamonti, A. E., Pincelli, A. I., Corra, B., Viarengo, R., Bonomo, S. M., Galimberti, D., . . . Muller, E. E. (2002). Plasma ghrelin concentrations in elderly subjects: comparison with anorexic and obese patients. *J Endocrinol, 175*(1), R1-5.

Santiago, Jcp, & Hallschmid, M. (2017). Central nervous insulin administration before nocturnal sleep decreases breakfast intake in healthy young and elderly subjects. *Frontiers in neuroscience, 11*(FEB) (no pagination). <http://onlinelibrary.wiley.com/o/cochrane/clcentral/articles/967/CN-01335967/frame.html> doi:10.3389/fnins.2017.00054

Sawaya, A. L., Fuss, P. J., Dallal, G. E., Tsay, R., McCrory, M. A., Young, V., & Roberts, S. B. (2001). Meal palatability, substrate oxidation and blood glucose in young and older men. *Physiol Behav, 72*(1-2), 5-12.

Schneider, Stéphane M, Al-Jaouni, Rima, Caruba, Céline, Giudicelli, Jean, Arab, Kamel, Suavet, Florence, . . . Hébuterne, Xavier. (2008). Effects of age, malnutrition and refeeding on the expression and secretion of ghrelin. *Clinical Nutrition, 27*(5), 724-731.

Toth, M. J., Arciero, P. J., Gardner, A. W., Calles-Escandon, J., & Poehlman, E. T. (1996). Rates of free fatty acid appearance and fat oxidation in healthy younger and older men. *J Appl Physiol (1985), 80*(2), 506-511.

Trahair, Laurence G, Horowitz, Michael, Rayner, Christopher K, Gentilcore, Diana, Lange, Kylie, Wishart, Judith M, & Jones, Karen L. (2012). Comparative effects of variations in duodenal glucose load on glycemic, insulinemic, and incretin responses in healthy young and older subjects. *The Journal of Clinical Endocrinology & Metabolism, 97*(3), 844-851.

Woolf, K., Reese, C. E., Mason, M. P., Beaird, L. C., Tudor-Locke, C., & Vaughan, L. A. (2008). Physical activity is associated with risk factors for chronic disease across adult women's life cycle. *J Am Diet Assoc, 108*(6), 948-959. doi:10.1016/j.jada.2008.03.015
