# Supplementary material for: Differences in circulating appetite-related hormone concentrations between younger and older adults: a systematic review and meta-analysis
Source: Aging Clin Exp Res. 2019 Aug 20;32(7):1233–44. doi: 10.1007/s40520-019-01292-6 (PMC7316693; doi:10.1007/s40520-019-01292-6)
Supplement: Supplementary file 7 — Supplementary material 7 (DOCX 33 kb) [file 40520_2019_1292_MOESM7_ESM.docx]

**Article Title:** Differences in circulating appetite-related hormone concentrations between older and younger adults: a systematic review and meta-analysis

**Journal:** Aging Clinical and Experimental Research

**Author Names:** Kelsie Olivia Johnson, Oliver Michael Shannon, Jamie Matu, Adrian Holliday, Theocharis Ispoglou, Kevin Deighton

**Corresponding Author:** Dr Kevin Deighton, Institute for Sport, Physical Activity & Leisure, Leeds Beckett University, Leeds, LS6 3QS, United Kingdom (email: K.Deighton@leedsbeckett.ac.uk)

**Supplementary Table 23.** AXIS Quality assessment for each included study

|  | 1 | 2 | 3 | 4 | 5 | 6 | 8 | 9 | 10 | 11 | 12 | 15 | 16 | 17 | 18 | 19 | 20 |
| --- | --- | --- | --- | --- | --- | --- | --- | --- | --- | --- | --- | --- | --- | --- | --- | --- | --- |
| Zambrano et al. 1996 | No | Yes | No | No | Do not know | Do not know | Yes | Yes | No | No | No | Yes | Yes | Yes | No | Yes | Do not know |
| Rzepka et al. | Yes | No | No | Yes | Yes | Yes | No | Yes | No | No | YEs | Yes | Yes | Yes | No | Do not know | Yes |
| Melanson et al. 1998 | Yes | Yes | No | No | Yes | Yes | Yes | Yes | No | No | Yes | Yes | Yes | Yes | No | Yes | Yes |
| Santiago *et al.* 2017 | Yes | Yes | No | Yes | Yes | Yes | Yes | Yes | Yes | Yes | No | No | No | No | No | Yes | Yes |
| Di Francesco et al. 2005 | Yes | Yes | No | Yes | Do not know | Do not know | Yes | Yes | No | No | Yes | Yes | Yes | Yes | Yes | Do not know | Yes |
| Moller *et al.* 1998 | No | Yes | No | Yes | Do not know | Do not know | Yes | Yes | No | No | Yes | No | No | No | No | Yes | Yes |
| Di Francesco et al. 2008 | Yes | Yes | No | Yes | Do not know | Do not know | Yes | Yes | No | No | Yes | Yes | Yes | No | Yes | No | Yes |
| Yukawa et al. 2006 | No | Yes | No | Yes | No | No | No | Yes | Yes | Yes | Yes | Yes | Yes | Yes | Yes | Yes | Yes |
| MacIntosh et al. 2001b | Yes | Yes | No | Yes | Yes | Do not know | Yes | Yes | Yes | No | No | Yes | No | Yes | No | Yes | Yes |
| MacIntosh et al. 2001a | Yes | Yes | No | No | Yes | Do not know | Yes | Yes | Yes | Yes | No | Yes | Yes | Yes | Yes | Do not know | Yes |
| MacIntosh et al. 1999 | Yes | No | No | No | Do not know | No | Yes | Yes | Yes | Yes | No | No | No | Yes | No | Do not know | Yes |
| Sturm et al. 2004 | Yes | Yes | No | Yes | Do not know | Do not know | Yes | Yes | Yes | Yes | Yes | Yes | No | Yes | No | No | Yes |
| Winkels et al. 200 | No | Yes | No | Yes | Yes | Yes | Yes | Yes | Yes | Yes | Yes | No | Yes | Yes | Yes | Yes | Yes |
| De La Maza | No | Do not know | No | Do not know | Do not know | Do not know | Yes | Yes | No |  | No | Yes | Yes | No | Yes | Yes | Yes |
| Bauer et al. 2010 | No | Yes | No | Yes | Yes | Yes | Yes | Yes | Yes | Yes | Yes | Do not know | Yes | No | No | Yes | Yes |
| Moss. 2012 | No | No | No | Yes | Yes | Yes | Yes | Yes | No | No | Yes | Yes | Yes | Yes | Yes | No | Yes |
| Bart et al. 2016 | Yes | Yes | No | No | Do not know | Do not know | Yes | Yes | Yes | Yes | Yes | Yes | Yes | Yes | Yes | Yes | Yes |
|  |  |  |  |  |  |  |  |  |  |  |  |  |  |  |  |  |  |
| Bertoli et al. 2006 | No | Yes | No | Yes | Do not know | Do not know | Yes | Yes | Yes | Yes | Yes | Yes | Yes | Yes | No | Do not know | Yes |
| Yukawa et al. 2008 | Yes | Yes | Yes | Yes | No | No | Yes | Yes | Yes | Yes | No | Yes | Yes | Yes | Yes | Do not know | Yes |
| Sawaya et al. 2001 | Yes | Yes | No | Yes | Do not know | Do not know | Yes | Yes | Yes | Yes | Yes | Yes | Yes | Yes | No | Yes | Yes |
| Woolf *et al.* 2008 | Yes | Yes | No | Yes | Yes | Yes | Yes | Yes | Yes | Yes | Yes | No | No | Yes | Yes | Yes | Yes |
| Rigamonti *et al.* 2002 | No | Yes | No | Yes | Do not know | Do not know | Yes | Yes | No | No | No | Yes | Yes | Yes | No | Yes | Yes |
| Berthélemy *et al.* 1992 | Yes | Yes | No | Yes | Yes | No | Yes | Yes | Yes | No | No | Yes | Yes | Yes | No | Do not know | Yes |
| Toth *et al.* 1996 | Yes | Yes | No | Yes | Yes | Yes | Yes | Yes | No | Yes | Yes | No | Yes | Yes | Yes | Yes | Yes |
| Ostlund *et al.* 1996 | No | Yes | No | Yes | Do not know | Do not know | Yes | Yes | Yes | No | Yes | No | No | No | Yes | Yes | Yes |
|  |  |  |  |  |  |  |  |  |  |  |  |  |  |  |  |  |  |
| Di Francesco *et al.* 2010 | No | Yes | Yes | Yes | Do not know | Do not know | Yes | Yes | Yes | Yes | No | Yes | No | Yes | Yes | Yes | Yes |
| Di Francesco *et al.* 2006 | Yes | Yes | No | Yes | Do not know | Do not know | Yes | Yes | Yes | Yes | No | Yes | Yes | Yes | Yes | Yes | Yes |
| Nass *et al.* 2014 | Yes | Yes | No | No | Do not know | Do not know | Yes | Yes | Yes | No | No | Yes | Yes | Yes | Yes | Yes | Yes |
|  |  |  |  |  |  |  |  |  |  |  |  |  |  |  |  |  |  |
| Trahair *et al.* 2012 | No | Yes | Yes | Yes | Do not know | Do not know | Yes | Yes | Yes | Yes | Yes | Yes | Yes | Yes | Yes | Yes | Yes |
| Schneider *et al.* 2008 | Yes | Yes | No | No | Do not know | Do not know | Yes | Yes | Yes | Yes | Yes | Yes | No | No | No | Yes | Yes |
| Khalil *et al.* 1985 | Yes | Yes | No | No | Do not know | Do not know | Yes | Yes | Yes | No | No | Yes | Yes | No | Yes | Yes | Yes |
|  |  |  |  |  |  |  |  |  |  |  |  |  |  |  |  |  |  |
| Franceschini *et al.* 1999 | No | Yes | No | Yes | Do not know | Do not know | Yes | Yes | No | No | Yes | Yes | Yes | No | No | Do not know | Yes |
| Flint *et al.* 2002 | Yes | Yes | Yes | Yes | Do not know | Do not know | No | Yes | Yes | Yes | No | Yes | Yes | Yes | No | Yes | Yes |
|  |  |  |  |  |  |  |  |  |  |  |  |  |  |  |  |  |  |
| Giezenaar *et al.* 2018a | No | No | No | No | Do not know | Do not know | Yes | Yes | Yes | No | No | Yes | Yes | Yes | Yes | Do not know | Yes |
| Giezenaar *et al.* 2018b | No | No | No | Do not know | Do not know | Do not know | Yes | Yes | Yes | No | Yes | Yes | Yes | Yes | Yes | Yes | Yes |

1 Were the aims/objectives of the study clear?

2 Was the study design appropriate for the stated aim(s)

3 Was the sample size justified?

4 Was the target/reference population clearly defined? (Is it clear who the research was about?)

5 Was the sample frame taken from an appropriate population base so that it closely represented the target/reference population under investigation

6 Was the selection process likely to select subjects/participants that were representative of the target/reference population under investigation?

7 Were measures undertaken to address and categorise non-responders?

8 Were the risk factor and outcome variables measured appropriate to the aims of the study?

9 Were the risk factor and outcome variables measured correctly using instruments/measurements that had been trialled, piloted or published previously?

10 Is it clear what was used to determined statistical significance and/or precision estimates? (e.g p-values, confidence intervals)

11 Were the methods (including statistical methods) sufficiently described to enable them to be repeated?

12 Were the basic data adequately described?

13 Does the response rate raise concerns about non-responders described?

14 If appropriate, was information about non-responders described?

15 Were the results internally consistent?

16 Were the results presented for all the analyses described in the methods?

17 Were the authors' discussion and conclusions justified by the results?

18 Were the limitations of study discussed?

19 Were there any funding sources or conflicts of interest that may affect the authors' interpretation of the results?

20 Was ethical approval or consent of participants attained?
