# Supplementary material for: Differences in circulating appetite-related hormone concentrations between younger and older adults: a systematic review and meta-analysis
Source: Aging Clin Exp Res. 2019 Aug 20;32(7):1233–44. doi: 10.1007/s40520-019-01292-6 (PMC7316693; doi:10.1007/s40520-019-01292-6)
Supplement: Supplementary file 8 — Supplementary material 8 (DOCX 199 kb) [file 40520_2019_1292_MOESM8_ESM.docx]

**Article Title:** Differences in circulating appetite-related hormone concentrations between older and younger adults: a systematic review and meta-analysis

**Journal:** Aging Clinical and Experimental Research

**Author Names:** Kelsie Olivia Johnson, Oliver Michael Shannon, Jamie Matu, Adrian Holliday, Theocharis Ispoglou, Kevin Deighton

**Corresponding Author:** Dr Kevin Deighton, Institute for Sport, Physical Activity and Leisure, Leeds Beckett University, Leeds, LS6 3QS, United Kingdom (email: K.Deighton@leedsbeckett.ac.uk)

**Supplementary Table 5.** Individual study statistics for studies evaluating postprandial acylated ghrelin concentrations

| Study | Hedges' g | Standard error | Variance | Lower limit | Upper limit | Z-Value | p-Value | Sample Size | | Weight |
| --- | --- | --- | --- | --- | --- | --- | --- | --- | --- | --- |
|  |  |  |  |  |  |  |  | Young | Old |  |
| Di Francesco et al. 2008 | -0.327 | 0.413 | 0.171 | -1.137 | 0.483 | -0.792 | 0.428 | 11 | 11 | 21.127 |
| Bauer et al. 2010 | 0.514 | 0.343 | 0.118 | -0.158 | 1.186 | 1.500 | 0.134 | 15 | 19 | 24.576 |
| Moss. 2012 | -0.108 | 0.455 | 0.207 | -1.000 | 0.785 | -0.236 | 0.813 | 18 | 6 | 19.259 |
| Nass et al. 2014 | -1.354 | 0.567 | 0.321 | -2.465 | -0.244 | -2.390 | 0.017 | 8 | 6 | 15.116 |
| Schneider et al. 2008 | -0.191 | 0.440 | 0.194 | -1.053 | 0.671 | -0.434 | 0.664 | 10 | 9 | 19.922 |
| Mean | -0.206 | 0.283 | 0.080 | -0.760 | 0.348 | -0.729 | 0.466 |  |  |  |

**Supplementary Table 6.** Individual study statistics for studies evaluating fasting total ghrelin concentrations

|  | Hedges' g | Standard error | Variance | Lower limit | Upper limit | Z-Value | p-Value | Sample Size | | Weight |
| --- | --- | --- | --- | --- | --- | --- | --- | --- | --- | --- |
|  |  |  |  |  |  |  |  | Young | Old |  |
| Bertoli et al. 2006 | 0.021 | 0.364 | 0.132 | -0.692 | 0.734 | 0.057 | 0.955 | 10 | 26 | 12.950 |
| Di Francesco et al. 2006 | -0.113 | 0.473 | 0.224 | -1.040 | 0.815 | -0.238 | 0.812 | 8 | 8 | 7.658 |
| Giezenaar et al. 2018 | 0.219 | 0.222 | 0.049 | -0.217 | 0.654 | 0.985 | 0.325 | 10 | 10 | 34.745 |
| Giezenaar et al. 2017 | -0.086 | 0.247 | 0.061 | -0.571 | 0.398 | -0.349 | 0.727 | 16 | 16 | 28.081 |
| Moss. 2012 | 0.395 | 0.459 | 0.210 | -0.504 | 1.294 | 0.861 | 0.389 | 18 | 6 | 8.147 |
| Schneider et al. 2008 | 0.646 | 0.451 | 0.204 | -0.239 | 1.530 | 1.431 | 0.152 | 10 | 9 | 8.420 |
| Mean | 0.132 | 0.131 | 0.017 | -0.124 | 0.389 | 1.011 | 0.312 |  |  |  |

**Supplementary Table 7.** Individual study statistics for studies evaluating postprandial total ghrelin concentrations

|  | Hedges' g | Standard error | Variance | Lower limit | Upper limit | Z-Value | p-Value | Sample Size | | Weight |
| --- | --- | --- | --- | --- | --- | --- | --- | --- | --- | --- |
|  |  |  |  |  |  |  |  | Young | Old |  |
| Yukawa et al. 2006 | 0.385 | 0.318 | 0.101 | -0.238 | 1.0073 | 1.211 | 0.226 | 21 | 18 | 13.740 |
| Bauer et al. 2010 | 0.161 | 0.338 | 0.114 | -0.501 | 0.8228 | 0.476 | 0.634 | 15 | 19 | 12.150 |
| Moss 2012. | 0.436 | 0.460 | 0.211 | -0.464 | 1.3370 | 0.950 | 0.342 | 18 | 6 | 6.567 |
| Bertoli et al. 2006 | -0.259 | 0.365 | 0.133 | -0.975 | 0.4565 | -0.710 | 0.478 | 10 | 26 | 10.400 |
| Di Francesco et al. 2006 | -0.003 | 0.473 | 0.224 | -0.929 | 0.9238 | -0.006 | 0.995 | 8 | 8 | 6.204 |
| Schneider et al. 2008 | 0.448 | 0.445 | 0.198 | -0.424 | 1.3197 | 1.007 | 0.314 | 10 | 9 | 7.005 |
| Giezenaar et al. 2018 | 0.207 | 0.256 | 0.065 | -0.294 | 0.7079 | 0.810 | 0.418 | 16 | 16 | 21.230 |
| Giezenaar et al 2017 | 0.097 | 0.247 | 0.061 | -0.388 | 0.5810 | 0.391 | 0.696 | 10 | 10 | 22.704 |
|  | 0.171 | 0.118 | 0.014 | -0.060 | 0.4020 | 1.454 | 0.146 |  |  |  |

**Supplementary Table 8.** Individual study statistics for studies evaluating fasting CCK concentrations

| Study | Hedges' g | Standard error | Variance | Lower limit | Upper limit | Z-Value | p-Value | Sample Size | | Weight |
| --- | --- | --- | --- | --- | --- | --- | --- | --- | --- | --- |
|  |  |  |  |  |  |  |  | Young | Old |  |
| Di Francesco et al. 2005 | 0.367 | 0.443 | 0.196 | -0.501 | 1.235 | 0.828 | 0.408 | 7 | 7 | 3.624 |
| Macintosh et al. 2001 | 0.190 | 0.166 | 0.028 | -0.135 | 0.516 | 1.145 | 0.252 | 9 | 9 | 25.749 |
| MacIntosh et al. 1999 | 0.236 | 0.357 | 0.128 | -0.464 | 0.937 | 0.662 | 0.508 | 8 | 8 | 5.566 |
| Sturm et al. 2004 | 0.347 | 0.235 | 0.055 | -0.113 | 0.808 | 1.477 | 0.140 | 12 | 12 | 12.879 |
| Winkels et al. 2011 | 0.713 | 0.357 | 0.127 | 0.014 | 1.412 | 1.999 | 0.046 | 15 | 17 | 5.590 |
| Bérthelemy et al. 1992 | 0.584 | 0.512 | 0.263 | -0.420 | 1.588 | 1.140 | 0.254 | 7 | 7 | 2.708 |
| Khalil et al. 1985 | 1.156 | 0.392 | 0.153 | 0.388 | 1.924 | 2.950 | 0.003 | 14 | 15 | 4.632 |
| Flint et al. 2002 | 0.370 | 0.159 | 0.025 | 0.059 | 0.681 | 2.333 | 0.020 | 20 | 20 | 28.222 |
| Giezenaar et al. 2017 | 0.666 | 0.254 | 0.064 | 0.168 | 1.163 | 2.622 | 0.009 | 16 | 16 | 11.030 |
|  | 0.407 | 0.084 | 0.007 | 0.242 | 0.573 | 4.830 | 0.000 |  |  |  |

**Supplementary Table 9.** Individual study statistics for studies evaluating postprandial CCK concentrations

|  | Hedges' g | Standard error | Variance | Lower limit | Upper limit | Z-Value | p-Value | Sample Size | | Weight |
| --- | --- | --- | --- | --- | --- | --- | --- | --- | --- | --- |
|  |  |  |  |  |  |  |  | Young | Old |  |
| Di Francesco et al. 2005 | 0.54 | 0.45 | 0.20 | -0.33 | 1.42 | 1.21 | 0.22 | 9 | 10 | 5.74 |
| Macintosh et al. 2001 | 0.24 | 0.17 | 0.03 | -0.09 | 0.56 | 1.42 | 0.16 | 12 | 12 | 34.17 |
| MacIntosh et al. 1999 | 0.18 | 0.36 | 0.13 | -0.52 | 0.88 | 0.50 | 0.62 | 7 | 8 | 8.86 |
| Sturm et al. 2004 | 0.42 | 0.29 | 0.08 | -0.14 | 0.99 | 1.48 | 0.14 | 12 | 12 | 13.29 |
| Winkels et al. 2011 | -0.04 | 0.35 | 0.12 | -0.71 | 0.64 | -0.11 | 0.91 | 15 | 17 | 9.43 |
| Bérthelemy et al. 1992 | 0.64 | 0.51 | 0.27 | -0.37 | 1.65 | 1.25 | 0.21 | 7 | 7 | 4.38 |
| Khalil et al. 1985 | 0.82 | 0.38 | 0.14 | 0.08 | 1.55 | 2.16 | 0.03 | 14 | 5 | 7.99 |
| Giezenaar et al. 2017 | 0.85 | 0.26 | 0.07 | 0.34 | 1.36 | 3.29 | 0.00 | 16 | 16 | 16.14 |
| Mean | 0.41 | 0.11 | 0.01 | 0.20 | 0.62 | 3.76 | 0.00 |  |  |  |

**Supplementary Table 10.** Individual study statistics for studies evaluating fasting GLP-1 concentrations

| Study | Hedges' g | Standard error | Variance | Lower limit | Upper limit | Z-Value | p-Value | Sample Size | | Weight |
| --- | --- | --- | --- | --- | --- | --- | --- | --- | --- | --- |
|  |  |  |  |  |  |  |  | Young | Old |  |
| MacIntosh et al. 2001 | -0.115 | 0.273 | 0.075 | -0.651 | 0.421 | -0.420 | 0.674 | 13 | 13 | 14.797 |
| MacIntosh et al. 1999 | 0.252 | 0.358 | 0.128 | -0.449 | 0.953 | 0.705 | 0.481 | 7 | 8 | 12.630 |
| Moss. 2012 | -0.232 | 0.456 | 0.208 | -1.126 | 0.663 | -0.507 | 0.612 | 18 | 6 | 10.337 |
| Di Francesco et al. 2010 | 0.218 | 0.285 | 0.081 | -0.340 | 0.776 | 0.765 | 0.444 | 12 | 12 | 14.499 |
| Trahair et al. 2012 | -0.592 | 0.207 | 0.043 | -0.997 | -0.186 | -2.859 | 0.004 | 12 | 12 | 16.499 |
| Giezenaar et al. 2018 | -0.460 | 0.224 | 0.050 | -0.900 | -0.020 | -2.051 | 0.040 | 10 | 10 | 16.063 |
| Giezenaar et al. 2017 | 0.880 | 0.259 | 0.067 | 0.372 | 1.387 | 3.397 | 0.001 | 16 | 16 | 15.175 |
| Mean | -0.016 | 0.214 | 0.046 | -0.435 | 0.404 | -0.073 | 0.942 |  |  |  |

**Supplementary Table 11.** Individual study statistics for studies evaluating postprandial GLP-1 concentrations

| Study | Hedges' g | Standard error | Variance | Lower limit | Upper limit | Z-Value | p-Value | Sample Size | | Weight |
| --- | --- | --- | --- | --- | --- | --- | --- | --- | --- | --- |
|  |  |  |  |  |  |  |  | Young | Old |  |
| Di Francesco et al. 2010 | 0.081 | 0.284 | 0.081 | -0.476 | 0.637 | 0.283 | 0.777 | 12 | 12 | 14.771 |
| Giezenaar et al. 2018 | -0.495 | 0.259 | 0.067 | -1.003 | 0.012 | -1.914 | 0.056 | 10 | 10 | 16.170 |
| Giezenaar et al. 2017 | 0.639 | 0.253 | 0.064 | 0.142 | 1.136 | 2.522 | 0.012 | 16 | 16 | 16.490 |
| Moss. 2012 | -0.031 | 0.455 | 0.207 | -0.923 | 0.861 | -0.067 | 0.946 | 18 | 6 | 8.213 |
| MacIntosh et al. 1999 | 0.156 | 0.357 | 0.127 | -0.543 | 0.855 | 0.437 | 0.662 | 7 | 8 | 11.419 |
| MacIntosh et al. 2001 | -0.066 | 0.273 | 0.075 | -0.601 | 0.470 | -0.240 | 0.810 | 13 | 13 | 15.356 |
| Trahair et al. 2012 | 0.382 | 0.235 | 0.055 | -0.080 | 0.843 | 1.622 | 0.105 | 12 | 12 | 17.580 |
| Mean | 0.109 | 0.153 | 0.023 | -0.190 | 0.409 | 0.716 | 0.474 |  |  |  |

**Supplementary Table 12.** Individual study statistics for studies evaluating fasting leptin concentrations

| Sample Size | Hedges' g | Standard error | Variance | Lower limit | Upper limit | Z-Value | p-Value | Sample Size | | Weight |
| --- | --- | --- | --- | --- | --- | --- | --- | --- | --- | --- |
|  |  |  |  |  |  |  |  | Young | Old |  |
| MacIntosh et al. 2001 | 0.234 | 0.234 | 0.055 | -0.224 | 0.693 | 1.001 | 0.317 | 12 | 12 | 10.789 |
| Di Francesco et al. 2006 | 0.397 | 0.478 | 0.228 | -0.540 | 1.334 | 0.831 | 0.406 | 30 | 28 | 11.096 |
| Bertoli et al. 2006 | 1.174 | 0.389 | 0.152 | 0.411 | 1.936 | 3.015 | 0.003 | 10 | 26 | 10.980 |
| De La Maza et al. 2007 | 2.082 | 0.424 | 0.180 | 1.251 | 2.914 | 4.907 | 0.000 | 31 | 10 | 11.018 |
| Franceschini et al. 1999 | -0.203 | 0.413 | 0.171 | -1.013 | 0.606 | -0.492 | 0.623 | 10 | 12 | 11.509 |
| Moller et al. 1998 | 0.350 | 0.289 | 0.084 | -0.217 | 0.917 | 1.211 | 0.226 | 23 | 24 | 11.383 |
| Ostlund et al. 1996 | -0.808 | 0.146 | 0.021 | -1.095 | -0.522 | -5.527 | 0.000 | 89 | 115 | 11.659 |
| Rzepka et al.2002 | 0.153 | 0.260 | 0.067 | -0.356 | 0.662 | 0.590 | 0.555 | 30 | 28 | 11.454 |
| Woolf et al. 2008 | 8.463 | 0.643 | 0.414 | 7.202 | 9.725 | 13.152 | 0.000 | 49 | 47 | 10.112 |
|  | 1.225 | 0.547 | 0.299 | 0.153 | 2.297 | 2.240 | 0.025 |  |  |  |

**Supplementary Table 13.** Individual study statistics for studies evaluating postprandial leptin concentrations

| Study | Hedges' g | Standard error | Variance | Lower limit | Upper limit | Z-Value | p-Value | Sample Size | | Weight |
| --- | --- | --- | --- | --- | --- | --- | --- | --- | --- | --- |
|  |  |  |  |  |  |  |  | Young | Old |  |
| Di Francesco et al. 2006 | 0.731 | 0.490 | 0.240 | -0.229 | 1.692 | 1.492 | 0.136 | 8 | 8 | 10.485 |
| Bauer et al.2010 | 0.226 | 0.338 | 0.114 | -0.437 | 0.890 | 0.669 | 0.504 | 15 | 19 | 15.709 |
| Bertoli et al. 2006 | 1.153 | 0.388 | 0.151 | 0.392 | 1.915 | 2.969 | 0.003 | 10 | 26 | 13.730 |
| Franceschini et al. 1999 | -0.100 | 0.412 | 0.170 | -0.908 | 0.707 | -0.244 | 0.807 | 12 | 10 | 12.880 |
| MacIntosh et al. 2001 | 0.259 | 0.234 | 0.055 | -0.200 | 0.718 | 1.106 | 0.269 | 12 | 12 | 20.574 |
| Schneider et al. 2008 | 1.170 | 0.478 | 0.229 | 0.233 | 2.107 | 2.447 | 0.014 | 10 | 9 | 10.815 |
| Yukawa et al. 2008 | 1.152 | 0.336 | 0.113 | 0.493 | 1.811 | 3.429 | 0.001 | 21 | 19 | 15.808 |
| Mean | 0.620 | 0.199 | 0.040 | 0.229 | 1.010 | 3.112 | 0.002 |  |  |  |

**Supplementary Table 14.** Individual study statistics for studies evaluating fasting insulin concentrations

| Study | Hedges' g | Standard error | Variance | Lower limit | Upper limit | Z-Value | p-Value | Sample Size | | Weight |
| --- | --- | --- | --- | --- | --- | --- | --- | --- | --- | --- |
|  |  |  |  |  |  |  |  | Young | Old |  |
| Di Francesco et al. 2006 | 0.241 | 0.136 | 0.019 | -0.026 | 0.509 | 1.770 | 0.077 | 8 | 8 | 3.794 |
| Di Francesco et al. 2010 | 0.244 | 0.140 | 0.019 | -0.029 | 0.518 | 1.750 | 0.080 | 12 | 12 | 5.611 |
| Zambrano et al. 1996 | 0.264 | 0.145 | 0.021 | -0.020 | 0.548 | 1.822 | 0.068 | 108 | 103 | 7.079 |
| Bertoli et al. 2006 | 0.197 | 0.133 | 0.018 | -0.063 | 0.457 | 1.485 | 0.138 | 10 | 26 | 4.599 |
| De La Maza et al. 2007 | 0.273 | 0.135 | 0.018 | 0.008 | 0.537 | 2.022 | 0.043 | 31 | 10 | 4.821 |
| Giezenaar et al. 2018a | 0.276 | 0.136 | 0.019 | 0.009 | 0.543 | 2.027 | 0.043 | 10 | 10 | 6.262 |
| Giezenaar et al. 2017 | 0.282 | 0.134 | 0.018 | 0.019 | 0.544 | 2.104 | 0.035 | 16 | 16 | 5.972 |
| Groen et al. 2016 | 0.246 | 0.137 | 0.019 | -0.023 | 0.515 | 1.792 | 0.073 | 12 | 12 | 4.491 |
| Moss. 2012 | 0.196 | 0.132 | 0.017 | -0.063 | 0.454 | 1.482 | 0.138 | 12 | 12 | 4.197 |
| MacIntosh et al. 2001a | 0.246 | 0.138 | 0.019 | -0.023 | 0.516 | 1.790 | 0.074 | 13 | 13 | 4.628 |
| MacIntosh et al. 2001b | 0.259 | 0.140 | 0.020 | -0.016 | 0.534 | 1.846 | 0.065 | 12 | 12 | 6.156 |
| Melanson et al. 1998 | 0.241 | 0.136 | 0.019 | -0.026 | 0.508 | 1.767 | 0.077 | 8 | 8 | 3.794 |
| Moller et al. 1998 | 0.237 | 0.139 | 0.019 | -0.036 | 0.510 | 1.701 | 0.089 | 23 | 24 | 5.571 |
| Rigamonti et al. 2002 | 0.189 | 0.129 | 0.017 | -0.065 | 0.442 | 1.458 | 0.145 | 12 | 7 | 3.460 |
| Sawaya et al. 2001 | 0.252 | 0.138 | 0.019 | -0.019 | 0.524 | 1.824 | 0.068 | 10 | 9 | 5.254 |
| Sturm et al. 2004 | 0.264 | 0.139 | 0.019 | -0.009 | 0.538 | 1.898 | 0.058 | 12 | 12 | 6.152 |
| Toth et al. 1996 | 0.222 | 0.138 | 0.019 | -0.048 | 0.493 | 1.610 | 0.107 | 18 | 30 | 5.470 |
| Trahair et al. 2012 | 0.135 | 0.108 | 0.012 | -0.076 | 0.346 | 1.254 | 0.210 | 12 | 12 | 6.243 |
| Woolf et al. 2008 | 0.222 | 0.140 | 0.020 | -0.053 | 0.498 | 1.585 | 0.113 | 49 | 47 | 6.445 |
| Mean | 0.237 | 0.132 | 0.017 | -0.022 | 0.495 | 1.793 | 0.073 |  |  |  |

**Supplementary Table 15.** Individual study statistics for studies evaluating postprandial insulin concentrations

| Study | Hedges' g | Standard error | Variance | Lower limit | Upper limit | Z-Value | p-Value | Sample Size | | Weight |
| --- | --- | --- | --- | --- | --- | --- | --- | --- | --- | --- |
|  |  |  |  |  |  |  |  | Young | Old |  |
| Santiago et al. 2017 | 0.693 | 0.293 | 0.086 | 0.118 | 1.267 | 2.361 | 0.018 | 32 | 19 | 6.679 |
| Bauer et al. 2010 | 0.691 | 0.347 | 0.121 | 0.010 | 1.372 | 1.988 | 0.047 | 15 | 9 | 4.925 |
| Bertoli et al. 2006 | 0.866 | 0.378 | 0.143 | 0.125 | 1.607 | 2.292 | 0.022 | 10 | 26 | 4.221 |
| Di Francesco et al. 2006 | 0.426 | 0.479 | 0.229 | -0.513 | 1.364 | 0.889 | 0.374 | 8 | 8 | 2.707 |
| Di Francesco et al. 2010 | 0.033 | 0.284 | 0.081 | -0.524 | 0.589 | 0.115 | 0.909 | 12 | 12 | 7.070 |
| Giezenaar et al 2018a | -0.343 | 0.257 | 0.066 | -0.846 | 0.160 | -1.336 | 0.182 | 10 | 10 | 8.413 |
| Giezenaar et al. 2017 | 0.021 | 0.247 | 0.061 | -0.463 | 0.505 | 0.085 | 0.932 | 16 | 16 | 8.986 |
| Groen et al. 2016 | 0.020 | 0.394 | 0.155 | -0.753 | 0.792 | 0.050 | 0.960 | 12 | 12 | 3.903 |
| Moss. 2012 | 0.347 | 0.397 | 0.158 | -0.432 | 1.126 | 0.873 | 0.383 | 12 | 12 | 3.845 |
| MacIntosh et al. 2001a | 0.073 | 0.380 | 0.144 | -0.672 | 0.818 | 0.191 | 0.848 | 13 | 13 | 4.178 |
| MacIntosh et al. 2001b | -0.191 | 0.234 | 0.055 | -0.649 | 0.267 | -0.818 | 0.413 | 12 | 12 | 9.852 |
| Melanson et al. 1998 | 0.059 | 0.284 | 0.081 | -0.498 | 0.615 | 0.207 | 0.836 | 8 | 8 | 7.068 |
| Nass et al. 2014 | 0.495 | 0.514 | 0.264 | -0.512 | 1.503 | 0.963 | 0.335 | 8 | 6 | 2.361 |
| Sawaya et al. 2001 | 0.176 | 0.319 | 0.102 | -0.449 | 0.801 | 0.552 | 0.581 | 10 | 9 | 5.760 |
| Schneider et al. 2008 | 0.231 | 0.440 | 0.194 | -0.632 | 1.095 | 0.525 | 0.599 | 10 | 9 | 3.169 |
| Sturm et al. 2004 | -0.031 | 0.284 | 0.081 | -0.588 | 0.525 | -0.110 | 0.913 | 12 | 12 | 7.070 |
| Trahair et al. 2012 | 0.307 | 0.235 | 0.055 | -0.153 | 0.766 | 1.307 | 0.191 | 12 | 12 | 9.793 |
| Mean | 0.163 | 0.081 | 0.007 | 0.005 | 0.321 | 2.022 | 0.043 |  |  |  |
|  |  |  |  |  |  |  |  |  |  |  |

**Supplementary Table 16.** Individual study statistics for studies evaluating fasting PYY concentrations

|  | Hedges' g | Standard error | Variance | Lower limit | Upper limit | Z-Value | p-Value | Sample Size | | Weight |
| --- | --- | --- | --- | --- | --- | --- | --- | --- | --- | --- |
|  |  |  |  |  |  |  |  | Young | Old |  |
| Di Francesco et al. 2005 | 0.006 | 0.439 | 0.193 | -0.854 | 0.867 | 0.015 | 0.988 | 9 | 10 | 22.941 |
| Giezenaar et al 2018a | -1.221 | 0.242 | 0.058 | -1.694 | -0.747 | -5.054 | 0.000 | 10 | 10 | 29.059 |
| Moss. 2012 | -0.092 | 0.455 | 0.207 | -0.985 | 0.800 | -0.202 | 0.840 | 18 | 6 | 22.424 |
| MacIntosh et al. 1999 | 0.083 | 0.356 | 0.127 | -0.615 | 0.782 | 0.234 | 0.815 | 7 | 8 | 25.577 |
| Mean | -0.353 | 0.382 | 0.146 | -1.102 | 0.397 | -0.922 | 0.357 |  |  |  |

**Supplementary Table 17.** Individual study statistics for studies evaluating postprandial PYY

|  | Hedges' g | Standard error | Variance | Lower limit | Upper limit | Z-Value | p-Value | Sample Size | | Weight |
| --- | --- | --- | --- | --- | --- | --- | --- | --- | --- | --- |
|  |  |  |  |  |  |  |  | Young | Old |  |
| Di Francesco et al. 2005 | 0.322 | 0.442 | 0.195 | -0.545 | 1.188 | 0.728 | 0.467 | 10 | 9 | 15.683 |
| Giezenaar et al 2018a | 0.546 | 0.260 | 0.067 | 0.037 | 1.055 | 2.104 | 0.035 | 10 | 10 | 45.431 |
| Moss. 2012 | 0.189 | 0.456 | 0.208 | -0.705 | 1.083 | 0.414 | 0.679 | 18 | 6 | 14.736 |
| MacIntosh et al.1999 | -0.061 | 0.356 | 0.127 | -0.760 | 0.637 | -0.173 | 0.863 | 7 | 8 | 24.151 |
| Mean | 0.312 | 0.175 | 0.031 | -0.031 | 0.655 | 1.781 | 0.075 |  |  |  |

**Supplementary Table 18.** Individual study statistics for studies evaluating fasting GIP concentrations

|  | Hedges' g | Standard error | Variance | | Lower limit | | Upper limit | Z-Value | p-Value | Sample Size | | Weight |
| --- | --- | --- | --- | --- | --- | --- | --- | --- | --- | --- | --- | --- |
|  |  |  | |  | |  |  |  |  | Young | Old |  |
| MacIntosh et al. 2001a | -0.008 | 0.273 | | 0.075 | | -0.543 | 0.527 | -0.029 | 0.976 | 13 | 13 | 22.345 |
| Trahair et al. 2012 | 0.377 | 0.204 | | 0.042 | | -0.023 | 0.778 | 1.847 | 0.065 | 16 | 16 | 27.608 |
| Giezenaar et al. 2018a | 0.531 | 0.225 | | 0.051 | | 0.089 | 0.973 | 2.356 | 0.018 | 10 | 10 | 25.926 |
| Giezenaar et al. 2018b | -0.346 | 0.249 | | 0.062 | | -0.833 | 0.142 | -1.389 | 0.165 | 12 | 12 | 24.121 |
| Mean | 0.157 | 0.196 | | 0.039 | | -0.228 | 0.541 | 0.798 | 0.425 |  |  |  |

**Supplementary Table 19.** Individual study statistics for studies evaluating postprandial GIP concentrations

|  | Hedges' g | Standard error | Variance | Lower limit | Upper limit | Z-Value | p-Value | Sample Size | | Weight |
| --- | --- | --- | --- | --- | --- | --- | --- | --- | --- | --- |
|  |  |  |  |  |  |  |  | Young | Old |  |
| Giezenaar et al 2017 | 0.2441 | 0.2479 | 0.0615 | -0.2417 | 0.7300 | 0.9848 | 0.3247 | 16 | 16 | 25.4791 |
| Giezenaar et al. 2018 | 0.9228 | 0.2684 | 0.0720 | 0.3967 | 1.4488 | 3.4380 | 0.0006 | 10 | 10 | 24.2054 |
| MacIntosh et al. 2001a | -0.0447 | 0.2732 | 0.0746 | -0.5802 | 0.4907 | -0.1638 | 0.8699 | 13 | 13 | 23.9119 |
| Trahair et al. 2012 | -0.0812 | 0.2333 | 0.0544 | -0.5384 | 0.3760 | -0.3481 | 0.7278 | 12 | 12 | 26.4036 |
| Mean | 0.2534 | 0.2264 | 0.0513 | -0.1903 | 0.6972 | 1.1194 | 0.2630 |  |  |  |

**Supplementary Table 20.** Individual study statistics for studies evaluating fasting hunger

| Study | Hedges’ g | Standard Error | Variance | Lower 5% Confidence Interval | Upper 95% Confidence Interval | z-value | p-value | Sample size | | | | Weight | | |
| --- | --- | --- | --- | --- | --- | --- | --- | --- | --- | --- | --- | --- | --- | --- |
|  |  |  |  |  |  |  |  | Young | Old | |  | | | |
| Santiago et al. 2017 | -1.065 | 0.509 | 0.259 | -2.063 | -0.068 | -2.094 | 0.036 | 8 | | 8 | 8.223 | | | |
| Di Francesco et al. 2005 | -0.410 | 0.444 | 0.197 | -1.280 | 0.460 | -0.924 | 0.356 | 9 | | 9 | 8.809 | | | |
| MacIntosh et al. 2001b | -1.657 | 0.461 | 0.213 | -2.561 | -0.754 | -3.595 | 0.000 | 12 | | 12 | 8.654 | | | |
| MacIntosh et al. 2001a | -0.310 | 0.382 | 0.146 | -1.059 | 0.439 | -0.811 | 0.417 | 13 | | 13 | 9.358 | | | |
| MacIntosh et al. 1999 | -1.633 | 0.571 | 0.326 | -2.752 | -0.513 | -2.859 | 0.004 | 7 | | 8 | 7.668 | | | |
| Sturm et al. 2004 | -0.438 | 0.399 | 0.159 | -1.221 | 0.344 | -1.098 | 0.272 | 12 | | 12 | 9.209 | | | |
| Winkels et al. 2011 | -0.270 | 0.347 | 0.120 | -0.950 | 0.410 | -0.779 | 0.436 | 15 | | 17 | 9.663 | | | |
| Moss. 2012 | -7.350 | 1.154 | 1.333 | -9.613 | -5.088 | -6.367 | 0.000 | 18 | | 6 | 3.816 | | | |
| Sawya et al. 2001 | -0.584 | 0.449 | 0.202 | -1.464 | 0.296 | -1.300 | 0.194 | 10 | | 9 | 8.763 | | | |
| Di Francesco et al. 2006 | -1.010 | 0.505 | 0.255 | -2.000 | -0.019 | -1.998 | 0.046 | 8 | | 8 | 8.255 | | | |
| Schneider et al. 2008 | -0.161 | 0.440 | 0.193 | -1.023 | 0.701 | -0.367 | 0.714 | 10 | | 9 | 8.847 | | | |
| Giezenaar et al. 2018 | -0.915 | 0.452 | 0.204 | -1.801 | -0.029 | -2.024 | 0.043 | 10 | | 10 | 8.735 | | | |
| Mean | -0.997 | 0.276 | 0.076 | -1.539 | -0.455 | -3.606 | 0.000 |  | |  |  | |  |  |

**Supplementary Table 21.** Individual study statistics for studies evaluating postprandial hunger

| Study | Hedges' g | Standard error | Variance | Lower limit | Upper limit | Z-Value | p-Value | Sample Size | | Weight |
| --- | --- | --- | --- | --- | --- | --- | --- | --- | --- | --- |
|  |  |  |  |  |  |  |  | Young | Old |  |
| Bauer et al | -0.259 | 0.339 | 0.115 | -0.923 | 0.405 | -0.765 | 0.444 | 15 | 19 | 14.232 |
| Di Francesco et al. 2005 | -0.352 | 0.443 | 0.196 | -1.219 | 0.516 | -0.795 | 0.427 | 9 | 10 | 10.077 |
| Di Francesco et al. 2006 | -0.971 | 0.503 | 0.253 | -1.957 | 0.015 | -1.931 | 0.053 | 8 | 8 | 8.360 |
| Moss. 2012 | -1.347 | 0.495 | 0.245 | -2.317 | -0.377 | -2.722 | 0.006 | 18 | 6 | 8.565 |
| MacIntosh et al 2001a | 0.060 | 0.273 | 0.075 | -0.475 | 0.596 | 0.220 | 0.826 | 13 | 13 | 17.870 |
| Schneider et al. 2008 | 0.456 | 0.445 | 0.198 | -0.416 | 1.329 | 1.025 | 0.305 | 10 | 9 | 9.997 |
| Sturm et al. 2018 | -0.429 | 0.287 | 0.083 | -0.992 | 0.134 | -1.492 | 0.136 | 12 | 12 | 17.014 |
| Winkels et al. 2011 | -0.160 | 0.346 | 0.120 | -0.838 | 0.518 | -0.464 | 0.643 | 15 | 17 | 13.885 |
| Mean | -0.308 | 0.167 | 0.028 | -0.636 | 0.020 | -1.839 | 0.066 |  |  |  |

**Supplementary Table 22.** Individual study statistics for studies evaluating energy intake

|  | Hedges' g | Standard error | Variance | Lower limit | Upper limit | Z-Value | p-Value | Sample Size | | Weight |  |  |  |  |  |  |  |  |  |  |
| --- | --- | --- | --- | --- | --- | --- | --- | --- | --- | --- | --- | --- | --- | --- | --- | --- | --- | --- | --- | --- |
|  |  |  |  |  |  |  |  | Young | Old |  |  |  |  |  |  |  |  |  |  |  |
| Giezenaar et al. 2018a | -0.074 | 0.222 | 0.049 | -0.508 | 0.361 | -0.332 | 0.740 | 10 | 10 | 10.153 |  |  |  |  |  |  |  |  |  |  |
| Giezenaar et al. 2017 | -2.005 | 0.304 | 0.092 | -2.601 | -1.409 | -6.596 | 0.000 | 16 | 16 | 9.597 |  |  |  |  |  |  |  |  |  |  |
| Moss. 2012 | -1.851 | 0.528 | 0.279 | -2.885 | -0.816 | -3.507 | 0.000 | 18 | 6 | 8.120 |  |  |  |  |  |  |  |  |  |  |
| MacIntosh et al. 1999 | -3.264 | 0.770 | 0.592 | -4.772 | -1.755 | -4.241 | 0.000 | 7 | 8 | 9.167 |  |  |  |  |  |  |  |  |  |  |
| MacIntosh et al. 2001a | -1.313 | 0.421 | 0.177 | -2.139 | -0.487 | -3.117 | 0.002 | 13 | 13 | 7.309 |  |  |  |  |  |  |  |  |  |  |
| MacIntosh et al. 2001b | 3.341 | 0.623 | 0.388 | 2.120 | 4.562 | 5.364 | 0.000 | 12 | 12 | 9.908 |  |  |  |  |  |  |  |  |  |  |
| Santigo et al. 2017 | 0.923 | 0.323 | 0.104 | 0.290 | 1.556 | 2.857 | 0.004 | 28 | 16 | 8.633 |  |  |  |  |  |  |  |  |  |  |
| Sturm et al. 2004 | -0.373 | 0.235 | 0.055 | -0.834 | 0.088 | -1.585 | 0.113 | 12 | 12 | 9.983 |  |  |  |  |  |  |  |  |  |  |
| Toth et al. 1996 | -6.587 | 0.733 | 0.538 | -8.024 | -5.149 | -8.981 | 0.000 | 18 | 30 | 7.509 |  |  |  |  |  |  |  |  |  |  |
| Woolf et al. 2008 | -0.757 | 0.210 | 0.044 | -1.168 | -0.345 | -3.607 | 0.000 | 49 | 47 | 9.950 |  |  |  |  |  |  |  |  |  |  |
| Zambrano et al. 1996 | -0.141 | 0.137 | 0.019 | -0.411 | 0.128 | -1.029 | 0.304 | 108 | 103 | 9.671 |  |  |  |  |  |  |  |  |  |  |
| Mean | -0.981 | 0.387 | 0.150 | -1.740 | -0.223 | -2.537 | 0.011 |  |  |  |  |  |  |  |  |  |  |  |  |  |
|  | | | | | | | | | | |  |  |  |  |  |  |  |  |  |  |
|  | | | | | | | | | | |  |  |  |  |  |  |  |  |  |  |
|  | | | | | | | | | | |  |  |  |  |  |  |  |  |  |  |
|  | | | | | | | | | | |  |  |  |  |  |  |  |  |  |  |
|  | | | | | | | | | | |  |  |  |  |  |  |  |  |  |  |
|  | | | | | | | | | | |  |  |  |  |  |  |  |  |  |  |
|  | | | | | | | | | | |  |  |  |  |  |  |  |  |  |  |
|  | | | | | | | | | | |  |  |  |  |  |  |  |  |  |  |
|  | | | | | | | | | | |  |  |  |  |  |  |  |  |  |  |
|  | | | | | | | | | | |  |  |  |  |  |  |  |  |  |  |
|  | | | | | | | | | | |  |  |  |  |  |  |  |  |  |  |
|  | | | | | | | | | | |  |  |  |  |  |  |  |  |  |  |
|  | | | | | | | | | | |  |  |  |  |  |  |  |  |  |  |
|  | | | | | | | | | | |  |  |  |  |  |  |  |  |  |  |
|  | | | | | | | | | | |  |  |  |  |  |  |  |  |  |  |
|  | | | | | | | | | | |  |  |  |  |  |  |  |  |  |  |
